# Cell Contractile Force‐Mediated Morphogenetic Tissue Engineering via 4D Printed Degradable Hydrogel Scaffolds

**DOI:** 10.1002/advs.202507288

**Published:** 2025-09-26

**Authors:** Aixiang Ding, Kaelyn L. Gasvoda, David S. Cleveland, Sriramya Ayyagari, Eben Alsberg

**Affiliations:** ^1^ Richard and Loan Hill Department of Biomedical Engineering University of Illinois at Chicago 909 S. Wolcott Ave. Chicago IL 60612 USA; ^2^ Jesse Brown Veterans Affairs Medical Center (JBVAMC) Chicago IL 60612 USA; ^3^ Departments of Mechanical & Industrial Engineering Orthopaedic Surgery, and Pharmacology and Regenerative Medicine University of Illinois at Chicago 909 S. Wolcott Ave. Chicago IL 60612 USA

**Keywords:** 4D printing, cell scaffold, hydrogel, shape morphing, tissue engineering

## Abstract

Tissue morphogenesis is a critical aspect of tissue development. Recent advances in 4D cell scaffolds have shown promise for modeling morphogenetic processes. While current 4D systems often rely on external stimuli, they frequently overlook the role of intrinsic cell‐generated forces, such as cell contractile forces (CCFs), in driving tissue morphogenesis. The paradox between the inherently weak nature of CCFs and the robustness of tissue scaffolds presents a significant challenge in achieving effective shape transformations. In this study, an easily printable, freestanding, cell‐laden hydrogel platform is designed to harness CCFs for 4D shape morphing. These hydrogels initially provide mechanical support to maintain structural integrity, followed by rapid degradation that amplifies CCFs through enhanced cell–cell interactions and increased local cell density, thereby inducing tissue morphogenesis. This platform enables the formation of scaffold‐free constructs with programmed shape transformations. By modulating the initial printed geometries, complex and large tissue constructs can be generated via controlled global shape transformations. Furthermore, the platform supports 4D tissue engineering by facilitating tissue differentiation coupled with dynamic shape evolution. This CCF‐4D system represents an important advancement in biomimetic tissue engineering, offering new avenues for creating dynamic tissue models that partially recapitulate native morphogenesis.

## Graphical abstract

Through the actuation of cell contractile forces, geometrically complex structures are formed via a bioprinted, rapidly degrading cell‐laden hydrogel system, resulting in cell‐only condensation within a few days of culture. This system supports stem cell differentiation down different lineages and new tissue formation, such as chondrogenesis and osteogenesis, while undergoing shape transformations simultaneously, demonstrating its potential for 4D tissue engineering applications.

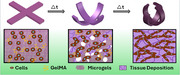

## 1 Introduction

Tissue morphogenesis, a critical aspect of biological development, has been widely studied due to its fundamental role in the formation of functional tissue structures.^[^ ^1^ ^]^ This process involves the organization of multicellular tissues into specific morphological architectures, driven by complex cellular interactions, including cell–cell and cell‐extracellular matrix (ECM) interactions.^[^ ^2^ ^]^ Such interactions are essential for achieving and maintaining tissue function, making the study of tissue morphogenesis highly relevant within the field of tissue engineering. Tissue engineering platforms provide a means to mimic and study tissue development in vitro,^[^ ^3^ ^]^ using models such as cell condensations^[^ ^3b^ ^]^ and hydrogel matrices^[^ ^4^ ^]^ to replicate tissue morphogenesis.

Cell condensation models are typically characterized by cell aggregation with initially minimal ECM components, forming tightly packed structures known as spheroids.^[^ ^5^ ^]^ Cell spheroids, characterized by strong cellular interactions^[^ ^3b^ ^]^ and rapid formation,^[^ ^5^, ^6^ ^]^ are frequently utilized for morphogenetic studies. However, these models have limited capacity to organize into more complex hierarchical structures, thereby restricting their ability to undergo dynamic morphogenetic processes such as bending, curling, folding, and buckling, which are critical to tissue development in vivo.^[^ ^1^, ^2^ ^]^ Conversely, conventional cell‐laden hydrogel models,^[^ ^7^ ^]^ while providing a more defined structure, often result in rigid constructs that limit cell–cell interactions and cellular rearrangement. These limitations hinder the replication of the dynamic curvature patterns observed in native tissue morphogenesis. Given these constraints, there is a need for alternative modeling strategies that better replicate the dynamic processes of tissue morphogenesis observed in vivo, including the complex curvature patterns formed through cell‐driven folding and bending.

4D biofabrication, an emerging approach in tissue engineering, incorporates time as an additional dimension, allowing for dynamic changes in scaffold shape to better mimic biological processes.^[^ ^7^, ^8^ ^]^ These geometric transformations were first observed in biological development and healing^[^ ^6^ ^]^ and have since been applied in various studies.^[^ ^8^, ^9^ ^]^ Methods for inducing shape changes in 4D scaffolds typically involve the fabrication of multi‐material systems with differential swelling^[^ ^8^, ^10^ ^]^ and employ various stimuli, including infrared light,^[^ ^11^ ^]^ temperature variations,^[^ ^12^ ^]^ electrical^[^ ^13^ ^]^ or magnetic fields,^[^ ^14^ ^]^ and chemical additives.^[^ ^15^ ^]^ However, these methods often rely on external stimuli, neglecting the intrinsic cellular forces that can play a role in driving tissue morphogenesis,^[^ ^16^ ^]^ which limits their ability to partially replicate this aspect of in vivo tissue formation processes.^[^ ^16a^ ^]^ Consequently, it may be valuable to develop more sophisticated 4D scaffolds that leverage endogenous biological mechanisms to replicate the dynamic changes of tissue architectures seen in living organisms.

Recent research has highlighted the critical role of cell contractile force (CCF) in driving complex tissue architecture formation in vivo.^[^ ^16^, ^17^ ^]^ Leveraging CCF to induce scaffold deformation presents a promising strategy for controlling 4D shape changes with potential clinical applications. Previous studies have explored CCF‐driven shape changes using engineered scaffolds, such as microplates,^[^ ^18^ ^]^ DNA Velcro,^[^ ^19^ ^]^ and photolithography‐assisted extrusion bioprinting.^[^ ^20^ ^]^ However, these methods face limitations in scalability, shape morphing resolution, and applicability in clinical settings due to challenges in achieving stable, large‐scale constructs and replicating the dynamic morphological changes required for in vivo‐like tissue formation. For example, 4D microplate structures that rely on cell seeding onto non‐biodegradable substrates encounter challenges in replicating the native 3D environments, where cells are embedded within the surrounding tissue matrices.^[^ ^18^ ^]^ DNA Velcro, another approach for achieving CCF‐mediated 4D shape changes, involves the deposition of loose cell aggregates onto superficial regions of a substrate.^[^ ^19^ ^]^ Although this method can successfully produce specific geometric changes, its reliance on complex procedures for precisely controlling the spatial positioning of cell aggregates presents a challenge for high throughput implementation. Extrusion bioprinting has been explored as a potential strategy for engineering large‐scale CCF‐based 4D tissue constructs with patterned cell distributions.^[^ ^20^ ^]^ However, the reported approach faces challenges related to limited shape‐morphing resolution over short timeframes, likely due to the softness of the materials, which makes it difficult to print stable, freestanding constructs. Consequently, current CCF‐driven systems are inadequate for achieving complex shape morphing on a large scale or replicating the dynamic 4D tissue formation observed in vivo. Additionally, the lack of gradual or controlled degradation of the scaffolds used further restricts their use in clinical applications and in vivo studies, as it impedes proper cell organization, rearrangement, and remodeling within the system. To address these challenges, there is a need to develop CCF‐driven actuation systems capable of generating stable, large tissue constructs without residual biomaterials that undergo 4D geometric changes to replicate the complex curvature patterns seen in native tissue morphogenesis.

**Figure 1:**
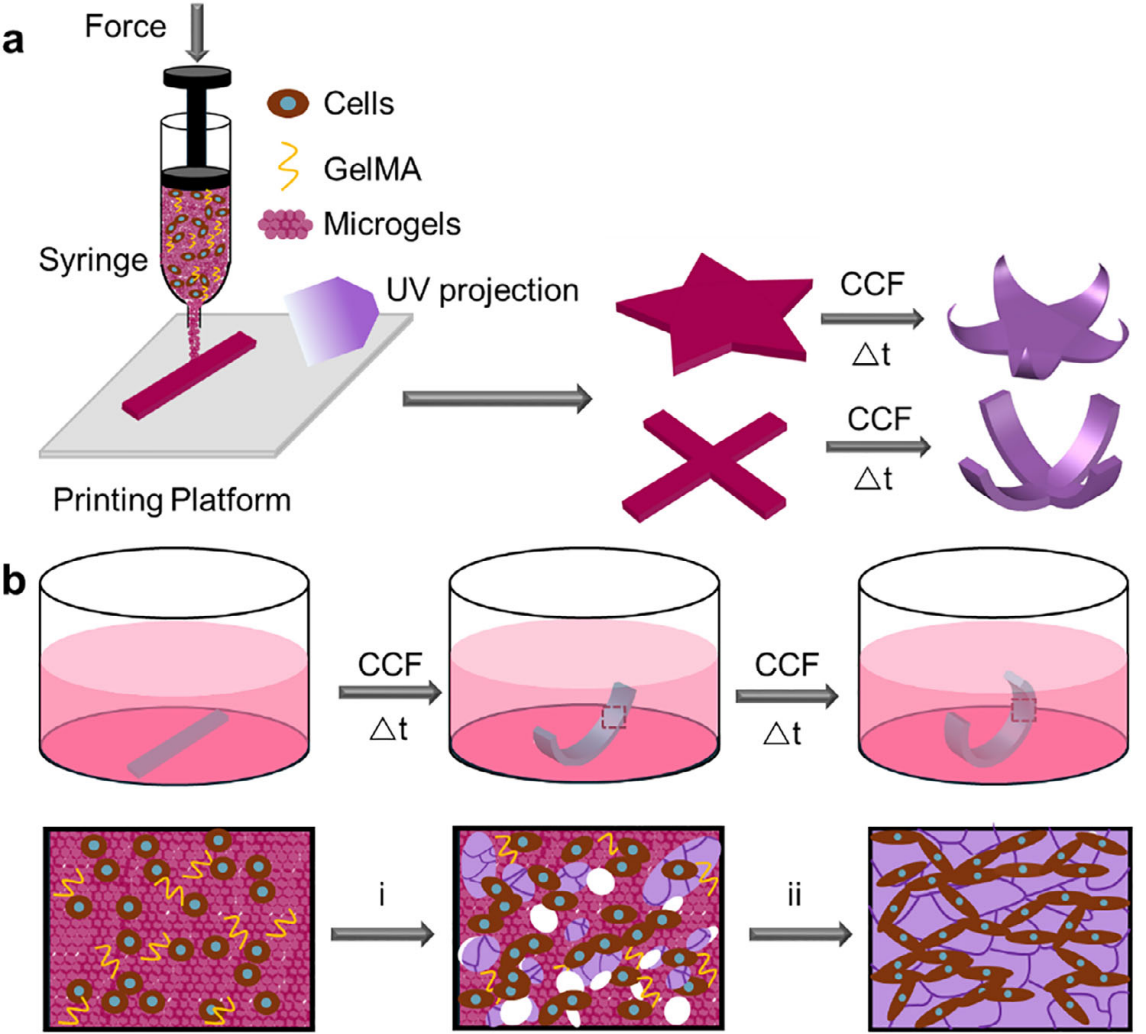
CCF‐mediated 4D bioprinting for the creation of geometrically transformable, scaffold‐free tissue constructs. a) Bioprinting of cell‐laden bioinks composed of living cells, GelMA, and OMA microgels, followed by CCF‐driven shape transformation. b) Dynamic shape morphing and tissue maturation of living constructs during culture. (i) Microgel degradation and tissue growth; (ii) Complete microgel degradation leading to the formation of tissue‐only (scaffold‐free) constructs.

In this study, we present a transformable, single‐material scaffold composed of a rapidly degradable hydrogel support structure fabricated via 4D bioprinting, which harnesses the inherent CCF exerted by encapsulated cells to achieve dynamic geometric transformations that partially replicate aspects of in vivo tissue morphogenesis (**Figure** **1**a). This scaffold consists of oxidized methacrylated alginate (OMA) microgels and cell‐adhesive gelatin methacrylate (GelMA), homogeneously embedded with live cells. Within this composite system, CCF drives scaffold deformation, leading to the formation of tissue‐only (scaffold material‐free) tissue constructs with predefined geometries following scaffold degradation and tissue maturation (Figure 1b). This approach offers significant advantages over existing methods by: 1) enabling the formation of more complex shapes and morphing patterns due to the high printability of the microgel bioink, which allows for the creation of freestanding constructs with high resolution and fidelity, and 2) utilizing CCF as a universal morphogenetic driver that supports long‐term 4D tissue formation. Our system thus provides a more biomimetic platform for replicating the specific geometries and morphologies of tissue development in vivo, with potential applications in regenerative medicine and tissue engineering.

## 2 Results and Discussion

### 2.1 Formulation of Hydrogel Bioinks and Rheological Characterization

To enable effective shape changes in the bioconstructs, the supporting hydrogel must be sufficiently flexible and mechanically weak to accommodate deformation driven by CCFs. A polymer composition that rapidly degrades within 3–4 days of post‐printing culture may be essential to achieve tissue‐only 4D condensation while preserving structural integrity during the initial printing process. Therefore, the design of an optimal hydrogel composition is critical.

OMA (Figure S1, Supporting Information) was synthesized as the base polymer for cell‐laden bioink formulation due to its photocrosslinkability, high cytocompatibility, and tunable degradability.^[^ ^21^ ^]^ OMA‐derived microgels with irregular morphology and an average diameter of 129 ± 68 µm (Figure S2, Supporting Information) were synthesized with rheological properties tailored for smooth extrusion printing.^[^ ^22^ ^]^ To enhance cell adhesion and interaction with the hydrogel matrix, a small amount of GelMA (3% w/v) (Figure S3, Supporting Information) was incorporated into the microgels. GelMA contains bioactive motifs, such as arginine‐glycine‐aspartic acid (RGD), which promote cell adhesion.^[^ ^23^ ^]^ After the printing of the composite bioinks, applying photoirradiation could stabilize the printed structures to form robust cell‐laden constructs suitable for transfer to and culture in media. It was hypothesized that, following rapid degradation of the OMA matrix, the scaffold would weaken, allowing cells within the construct to exert CCFs and drive shape morphing. This shape change would be facilitated by both the proliferation of cells and the resulting increase in CCFs. Consequently, the composite bioink formulation consisting of OMA microgels and GelMA (OMAGM) was developed for use in this study.

**Figure 2:**
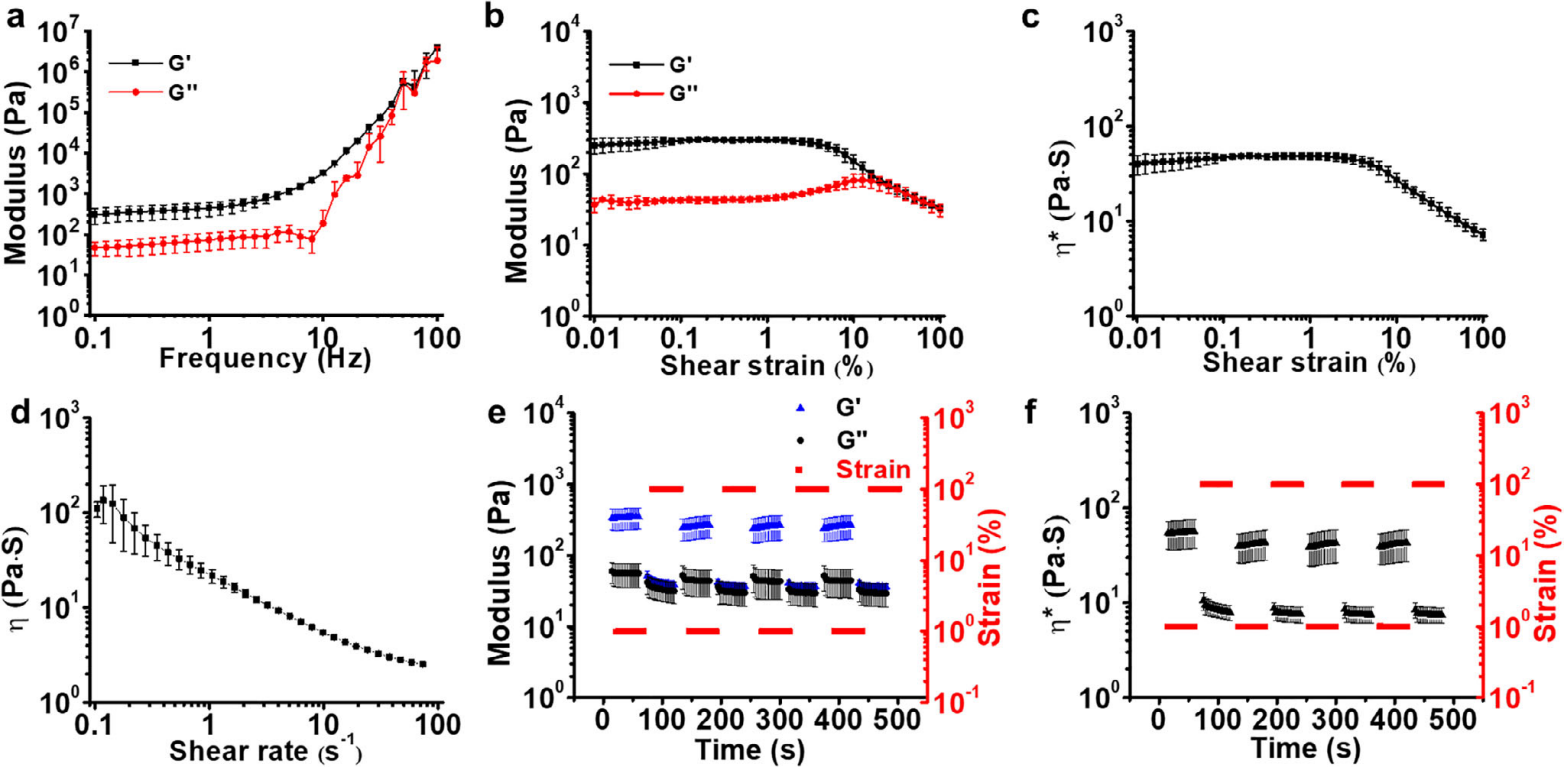
Rheological properties of the OMAGA bioink. a) Storage modulus (G') and loss modulus (G") as a function of frequency. b) Changes in G' and G“ with increasing shear strain. c) Complex viscosity (η^*^) as a function of shear strain. d) Viscosity (η) as a function of shear rate. e) Modulus and f) complex viscosity changes over time under cyclic shear strain of 1% and 100%.

To successfully print freestanding 3D constructs, the composite bioink must exhibit solid‐like behavior for structural stability while also possessing shear‐thinning properties to ensure smooth extrusion. To evaluate these characteristics, rheological tests were performed on the bioink. A frequency sweep test at 0.1% strain revealed a significantly higher storage modulus (G′) than loss modulus (G″), indicating a predominantly solid‐like structure at low frequencies (e.g., 0.1–40 Hz) (**Figure** **2**a). To further assess the shear‐thinning properties, strain sweep and shear rate ramp tests were conducted. The results showed that G′ remained greater than G″ across most of the applied strain range, confirming that the bioink retained its structural integrity at low strains (Figure 2b). A crossover between G′ and G″ at 20% strain marked the transition from a solid‐like to a liquid‐like state, demonstrating the material's ability to flow under higher strain conditions. Further analysis of the phase transition was evident from the complex viscosity‐shear strain and viscosity‐shear rate graphs. The complex viscosity‐shear strain curve showed a gradual decrease in viscosity beginning at 4% strain (Figure 2c), while the viscosity‐shear rate curve demonstrated a consistent reduction in viscosity with increasing shear rate (Figure 2d). By applying a Power‐Law fit to the log‐log plot of viscosity versus shear rate within the linear range^[^ ^24^ ^]^ from 0.22 to 2.07 s^−1^, we determined the flow index (*n*) and the consistency index (*K*) to be 0.26 and 21.66, respectively. These results confirm the bioink's shear‐thinning behavior, which is critical for ensuring sufficient flowability during extrusion.

Self‐healing is another key parameter for maintaining the stability of printed constructs. After printing, the OMAGM bioink must quickly recover its initial structural state to maintain a stable freestanding form. To evaluate the self‐healing ability, a cyclic shear strain test alternating between 1% and 100% strain was conducted. The high strain simulated the shear forces encountered during extrusion, while the low strain represented the post‐extrusion state. As shown in Figure 2e,f, the bioink exhibited a rapid decrease in modulus and complex viscosity under high strain, followed by an immediate recovery when the strain was released. Minimal hysteresis between cycles indicated that the OMAGM bioinks quickly regained mechanical stability.^[^ ^25^ ^]^ Even after four cycles, the OMAGM bioink demonstrated consistent recovery capability. Similar rheological performance was observed with the OMA‐only bioinks (Figure S4, Supporting Information). These results confirm the suitability of both OMAGM and OMA‐only bioinks for extrusion‐based printing.

After confirming the favorable rheological properties for 3D printing, the printability of the bioinks into hydrogel constructs was further evaluated using the parameters detailed in Table S1 (Supporting Information). Extrusion through a 22‐gauge needle (inner diameter: 413 µm) produced uniform filaments (Figure S5a, Supporting Information) with high printing fidelity as demonstrated by dimensional accuracy ranging from 101% to 106% across the x, y, and z axes (Figure S5b, Supporting Information). Large hydrogel constructs with various geometries, including pyramids, cubes, and cylinders (Figure S5c, Supporting Information), as well as pyramids of increasing heights from 4  to 8 mm (Figure S5d, Supporting Information), were printed with excellent structural stability both before and after UV crosslinking. These results confirm the robust printability and structural integrity of the OMAGM bioinks.

### 2.2 Mechanical Characteristics and Degradation of 3D Printed Hydrogel Constructs

**Figure 3:**
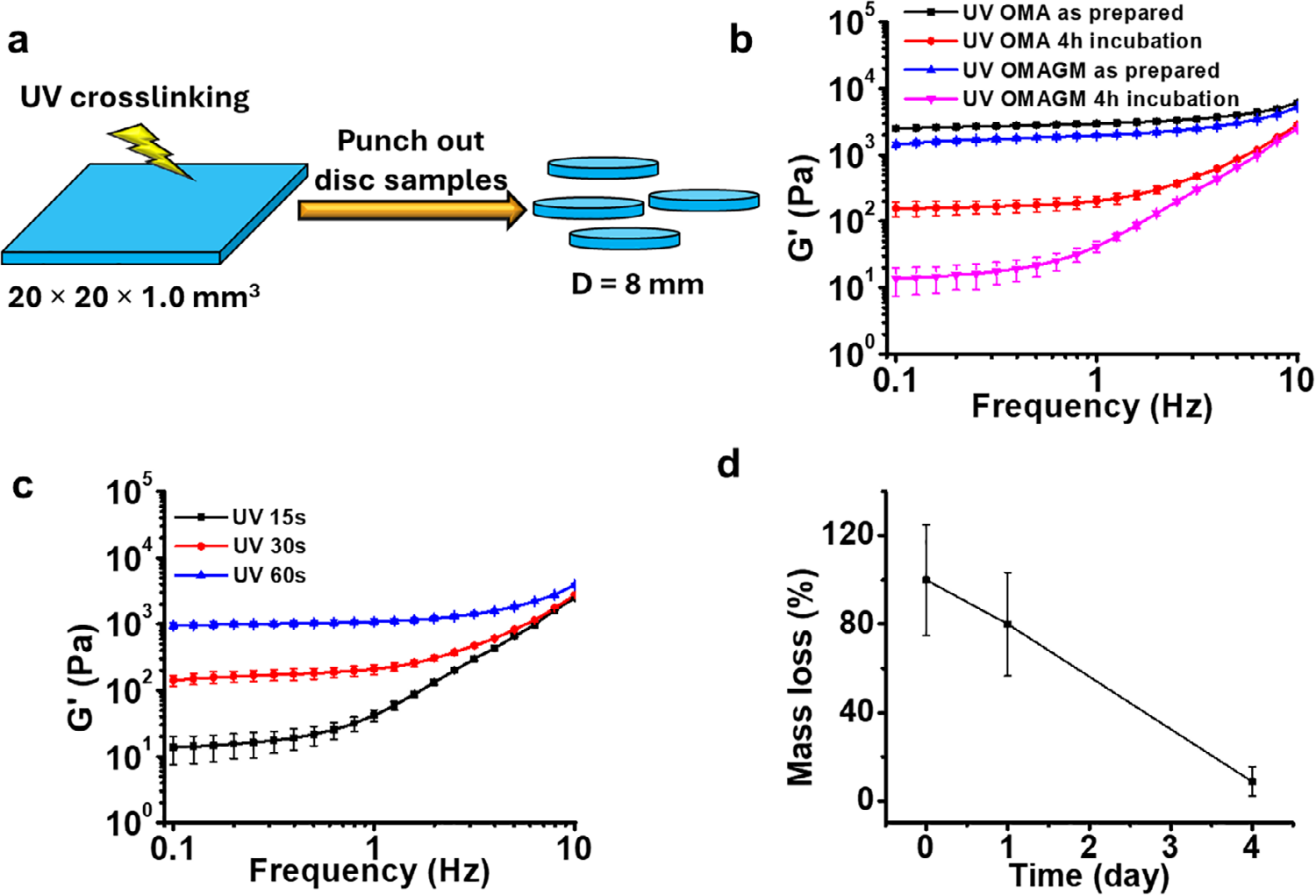
Mechanical properties and degradation behavior of hydrogel constructs. a) Schematic illustration of hydrogel disc preparation. b) Storage modulus (G') of as‐prepared and swollen OMA and OMAGM hydrogels as a function of frequency. c) Storage modulus (G') of swollen OMAGM hydrogels after varying UV crosslinking time. d) Mass loss profile of OMAGM hydrogels with a UV crosslinking time of 15 s.

To characterize the mechanical and degradation properties of the printed hydrogel constructs, a hydrogel slab measuring 20 × 20 × 1.0 mm (L × W × H) was initially printed, photocrosslinked (15 s, 6.7 mW cm^−^^2^), and then punched into multiple hydrogel discs (d = 8 mm) for testing (**Figure** **3**a). These samples were subjected to dynamic mechanical analysis using frequency sweep mode. The rheogram in Figure 3b shows that both as‐prepared OMA and OMAGM hydrogels exhibited a frequency‐independent storage modulus (G'), which is a typical feature of chemically crosslinked hydrogels.^[^ ^26^ ^]^ The OMAGM hydrogel had a lower G' than the OMA hydrogel, suggesting that incorporating GelMA into the OMA matrix resulted in a softer hydrogel.

After incubation in cell‐expansion media at 37 °C for 4 h, both OMAGM and OMA‐only hydrogels reached a swollen state, which resulted in a significant decrease in G' and a shift to strong frequency dependence due to network weakening. The swollen OMAGM hydrogel exhibited a much lower G' compared to the swollen OMA hydrogel, indicating a weaker matrix due to GelMA incorporation. This reduction in hydrogel stiffness is advantageous for facilitating CCFs to induce deformation. While the as‐prepared hydrogels possessed sufficient mechanical rigidity for handling and transferring into culturing media, the marked decrease in stiffness upon swelling is particularly favorable for cell‐mediated contraction. Notably, the G' of the swollen OMAGM hydrogel decreased to below 200 Pa, a value lower than the elastic modulus of most soft tissues,^[^ ^27^ ^]^ thereby reducing mechanical resistance and enabling CCFs to more effectively contract the hydrogel matrix.

The effect of UV crosslinking time on the G' of the swollen OMAGM hydrogels was further investigated. Increasing UV crosslinking time enhanced chemical crosslinking, substantially increasing hydrogel stiffness (Figure 3c). A UV crosslinking time of 15 s yielded a weak yet structurally sound hydrogel matrix, with a G' below 20 Pa, which was chosen for stabilizing the printed construct for further studies. Degradation tests revealed that OMAGM hydrogels fabricated with 15 s of UV crosslinking underwent rapid degradation within 4 days of culture in media (Figure 3d), whereas extending the UV exposure time slowed the degradation rate (Figure S6, Supporting Information). This rapid degradation not only weakens the hydrogel matrix but also creates space for enhanced cell–cell interactions. Previous study has shown that such interactions can significantly increase cell‐traction forces (or CCFs).^[^ ^28^ ^]^ Therefore, rapid degradation may enhance the ability of CCFs to contract the hydrogel matrix, acting as a key mechanism for construct deformation and the formation of scaffold‐free structures.

### 2.3 Examination of the Roles of Live Cells in Driving 4D Shape Transformation

**Figure 4:**
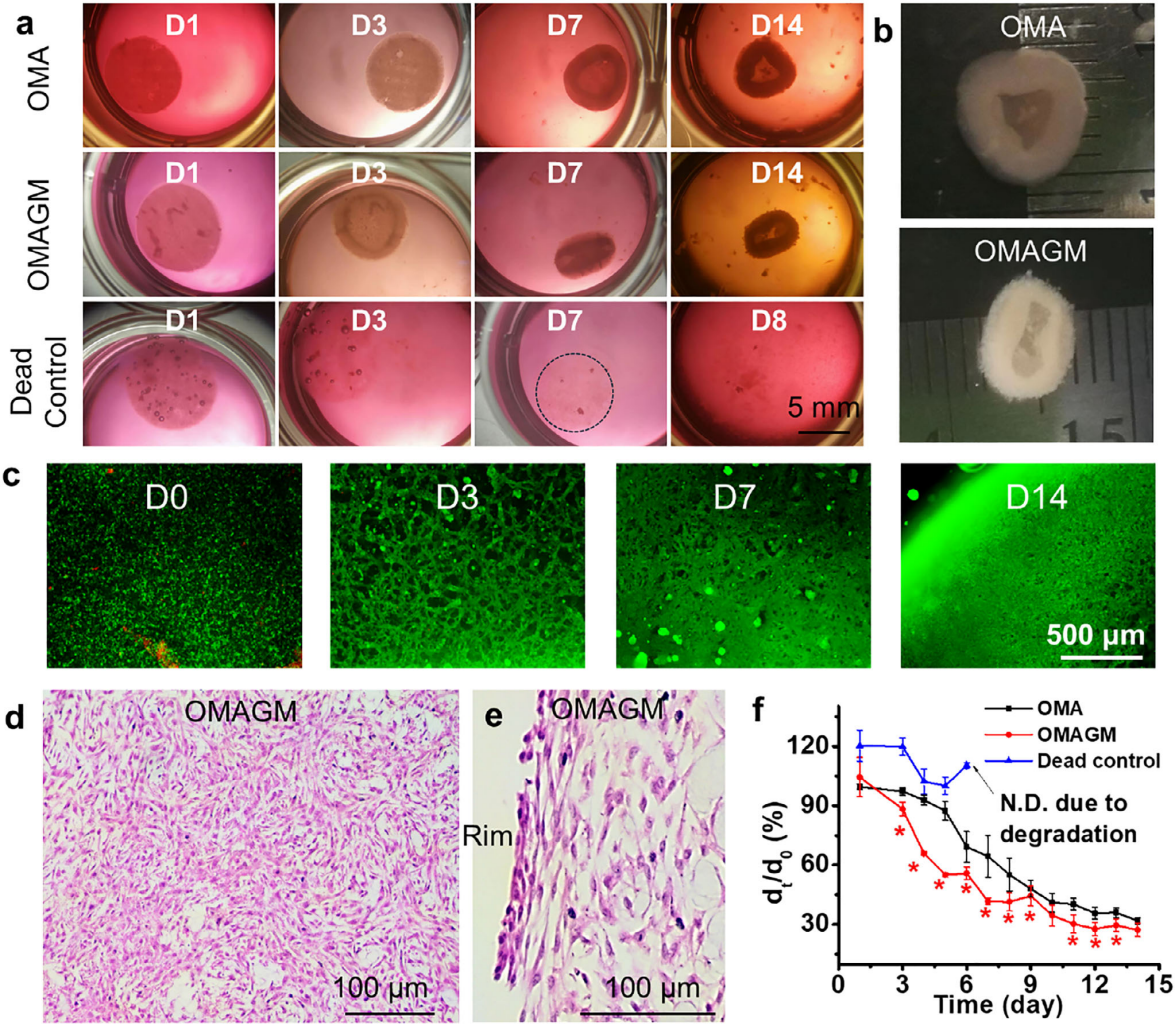
Roles of live cells in driving 4D shape transformation. a) Time‐lapse images of various cell‐laden constructs on 1, 3, 7, and 14 days of culture. Note that the dead cell constructs disintegrated completely by D7 and are not shown thereafter, with only loose polymer fragments remaining in the dish. b) Morphed tissue‐only constructs derived from the OMA and OMAGM groups. c) Live/dead staining images of cell‐laden OMAGM constructs at different times. d,e) H&E staining images of the tissue‐only construct formed from the OMAGM group. f) Change in diameter ratio (d_t_/d_0_) over culture time, where d_t_ is the diameter of the disc construct on a given day, and d_0_ represents the initial diameter on D0 (8 mm). ^*^ *p* < 0.05 compared to the groups of OMA and dead control. Construct fabrication parameters: cell density: 100 M cells mL^−1^; UV: 15s at 20 mW cm^−2^; disc dimensions: d_0_ = 8.0 mm, h = 1.0 mm.

In soft hydrogels like collagen, live cells have been shown to induce effective matrix contraction through CCFs, with fibroblasts identified as a robust cell type for these studies.^[^ ^29^ ^]^ In this study, the role of living cells in driving 4D shape transformation via controlled matrix contraction was investigated using NIH3T3 fibroblasts as a model, due to their rapid proliferation and documented capacity to contract hydrogel constructs.^[^ ^30^ ^]^ Cell‐laden hydrogel constructs using OMA and OMAGM bioinks were printed. The experimental setup included three groups of NIH3T3‐laden hydrogels, each with a cell density of 1 × 10^8^ (100 M) cells mL^−1^ bioink: OMA, OMAGM, and an OMAGM loaded with dead cells serving as a control. Disc‐shaped constructs, prepared as illustrated in Figure 3a, were cultured in cell‐growth media for 14 days. Live/dead staining conducted 4 h post‐fabrication indicated high cell viability within OMA and OMAGM constructs, whereas cells in the dead control constructs were completely nonviable (Figure S7, Supporting Information). Visually, the OMA and OMAGM disc constructs containing live cells progressively curled upward to form bowl‐shaped structures (**Figure** **4**a). In contrast, the dead control and cell‐free OMA and OMAGM (Figure S8, Supporting Information) constructs maintained a flat shape throughout the culture period until they eventually collapsed due to hydrogel degradation. The OMA and OMAGM constructs with live cells remained structurally integrated over the 14 days, forming well‐morphed constructs (Figure 4b). Live/dead staining revealed high cell viability, a marked increase in cell density, and the formation of dense cellular networks in these constructs as the culture period extended from D0 to D14 (Figure 4c). H&E staining over 14 days of culture revealed that cells within the OMAGM constructs progressively formed into well‐organized structures (Figure 4d; Figure S9, Supporting Information), with no residual hydrogel visualized at D14, indicating that only cells and ECM remained. Interestingly, the cells at the construct rims appeared more densely organized, potentially contributing to differential contraction along the radial direction and, consequently, the observed curling (Figure 4e).

We then quantified the deformation and outlined the deformation profiles of the three groups, following the protocols described in Figure S10 (Supporting Information), with results shown in Figure 4f. The quantified deformation data aligned with visual observations, showing more rapid and pronounced contraction in OMAGM constructs compared to OMA. In contrast, constructs containing dead cells exhibited volumetric expansion rather than contraction, likely due to the absence of effective cell‐matrix interactions and CCFs. Data collection beyond D4 was not feasible for this group, as the constructs underwent substantial degradation. Previous studies on cell‐laden collagen hydrogels showed that the primary mechanism driving hydrogel contraction is the CCFs exerted on the matrix during cell attachment and locomotion.^[^ ^31^ ^]^ The presence of GelMA in OMAGM likely facilitates stronger initial cell adhesion to the hydrogel matrix, enabling better transfer of CCFs and more significant and rapid shape deformation than in OMA constructs. Conversely, in the dead control samples (dead cell‐laden OMAGM), the inability of dead cells to adhere to the hydrogel or secrete ECM led to rapid construct degradation and disintegration within a week. Interestingly, the degradation of these dead cell samples was slower than that of empty OMAGM constructs (Figure 3d), possibly due to the large cell quantity within them.

Previous studies on cell‐induced contraction in soft collagen hydrogels have shown homogeneous matrix contraction, likely due to the large pore sizes in collagen hydrogels, which allow easy reorganization by cell traction and locomotion.^[^ ^32^ ^]^ Consequently, only in‐plane shrinkage of construct shapes was observed in those studies. In this study, the mechanically sound yet degradable OMAGM matrix provided temporary mechanical resistance, inducing anisotropic contraction during the initial days of culture. This facilitated observable deformation, underscoring the crucial role of a structurally stable but degradable hydrogel matrix in designing CCF‐inducible, shape‐transformable hydrogel constructs. However, the specific mechanisms underlying this anisotropic contraction remain unclear at this stage. One possible explanation is that hydrogel regions closer to the edge exhibited a faster degradation profile due to greater accessibility to the culture media, leading to enhanced media exchange and accelerated hydrolysis. Consequently, cells in these regions generated stronger collective contractile forces, inducing anisotropic contraction along radial directions. Along with the restriction imposed by the well plate at the bottom, this contraction resulted in the hydrogel disc curling upward into a bowl‐like shape. While GelMA accelerates shape deformation, OMA, despite lacking active cell‐adhesive bio‐ligands, undergoes rapid degradation, generating pores that weaken the mechanical integrity of the matrix and facilitate cell–cell interactions. Consequently, effective shape deformation was also observed in cell‐laden OMA‐only constructs.

### 2.4 F‐Actin and E‐Cadherin Staining

CCFs are primarily generated by the actin‐myosin cytoskeletal machinery within cells. This process involves the formation of actomyosin complexes through the interactions between filamentous actin (F‐actin) and myosin II motor proteins, which produce tension via ATP hydrolysis.^[^ ^33^ ^]^ Through this mechanism and physical coupling with surrounding cells and ECM/materials, cells can exert traction forces on their environment, activating mechanotransduction pathways that regulate behaviors such as proliferation, migration, and lineage‐specific differentiation.^[^ ^17^ ^]^ This force‐mediated communication with the cell microenvironment plays a pivotal role in tissue morphogenesis and structural remodeling.

**Figure 5:**
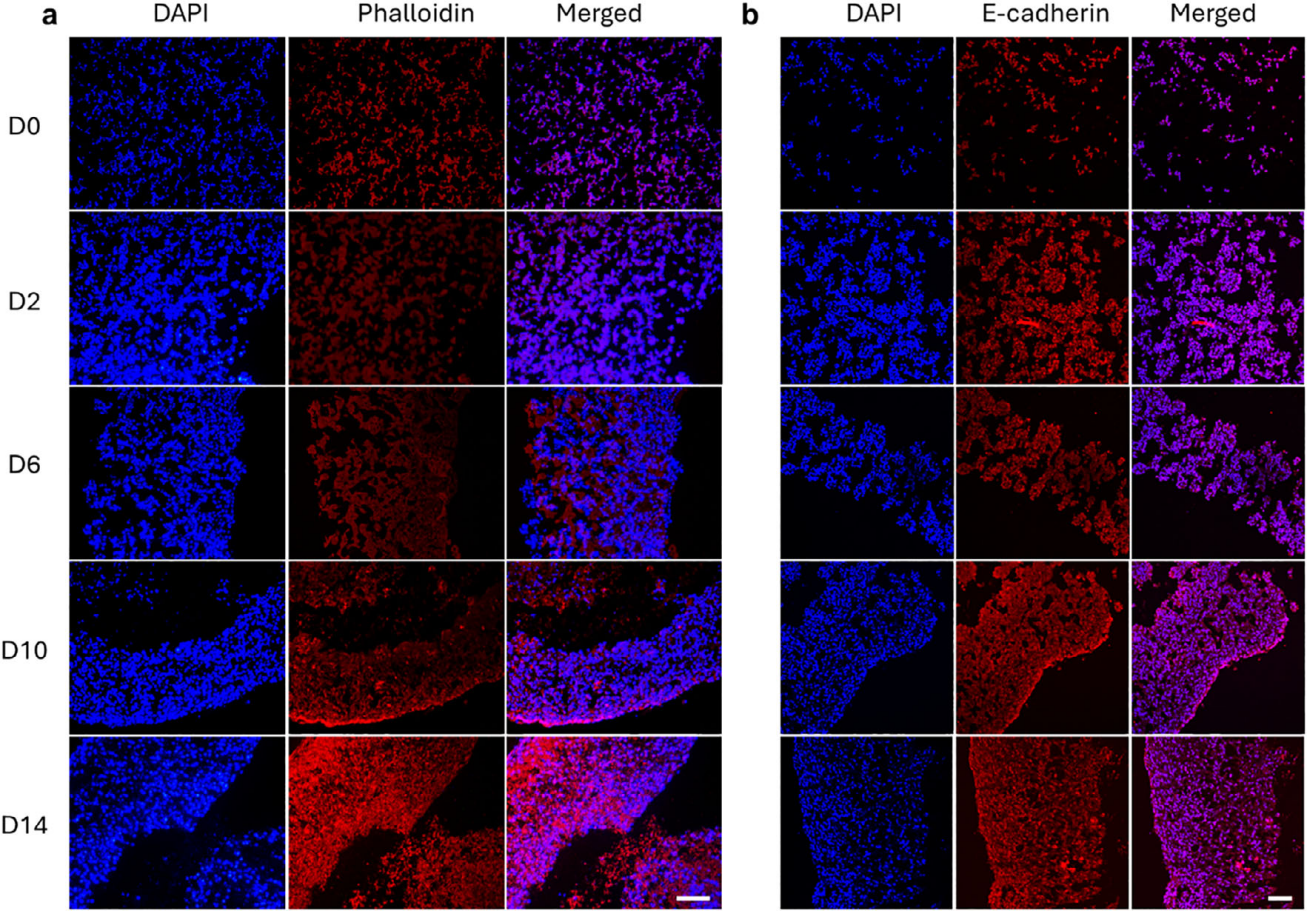
Immunofluorescence staining of cytoskeletal and adhesion markers in cell‐laden OMAGM constructs at different culture time points. a) Phalloidin staining for F‐actin and b) immunostaining for E‐cadherin revealing progressive cytoskeletal organization and cell–cell adhesion over time. Scale bars = 100 µm. Construct fabrication parameters: cell density: 100 M cells mL^−1^; UV: 15s at 20 mW cm^−2^; disc dimensions: d_0_ = 8.0 mm, h = 1.0 mm.

To visualize the organization and development of actin cytoskeleton networks, phalloidin staining, which specifically binds to F‐actin and enables fluorescent imaging of cytoskeletal architecture, was performed. OMAGM constructs encapsulating live cells were harvested at various culture time points and subjected to phalloidin staining (**Figure** **5**a). At D0, cells appeared round and evenly dispersed, with weak and disorganized F‐actin staining. As culture progressed over time, a gradual increase in cell density, a transition from round to elongated morphologies, and emergence of well‐aligned cellular arrangements were observed. Notably, at later time points such as D10 and D14, strong and polarized F‐actin staining was evident, particularly at the construct periphery, indicating potentially elevated anisotropic CCF generation and coordinated cytoskeletal remodeling. The observed F‐actin organization patterns were consistent with histological features revealed by H&E staining (Figure 4d,e; Figure S9, Supporting Information). This cellular organization evolved in concert with the macroscopic shape morphing of the constructs.

In parallel, cell–cell adhesion was assessed by immunofluorescent staining for E‐cadherin (Figure 5b), a key membrane‐bound protein involved in adherens junctions. E‐cadherin‐mediated adhesion, in concert with the actin cytoskeleton, contributes to force transmission across cells and facilitates collective migration and morphogenetic patterning.^[^ ^34^ ^]^ Initially, cells displayed sparse E‐cadherin expression and a scattered distribution. Over time, expression intensified and became more localized at the tissue edges, paralleling the alignment and compaction of cells into network‐like structures by D10 and D14. These findings further support the role of coordinated intercellular adhesion in collective force generation and tissue morphodynamics.

Together, the dynamic reorganization of F‐actin and E‐cadherin over time reflects the emergence of structured mechanical anisotropy within the constructs. CCFs, regulated by the F‐actin and E‐cadherin machinery, are transmitted to the surrounding ECM and neighboring cells, driving global hydrogel deformation and contributing to the observed 4D shape morphing. Therefore, modulating actin polymerization or cadherin expression could thus serve as a powerful strategy to investigate and direct tissue morphogenesis in vitro.

### 2.5 Impact of Cell Density, Hydrogel Stiffness, and Construct Dimensions on Shape‐Morphing Capability

Having established the critical role of CCFs in driving the shape transformation of these cell‐laden constructs, the effects of cell density, hydrogel stiffness, and construct dimensions on shape‐morphing were investigated. Initially, cell densities ranging from 20 to 200 M cells mL^−1^ of bioink were incorporated into OMAGM hydrogel constructs. Constructs were subjected to live/dead staining after 3 h of culture to assess cell viability. Cells within the constructs remained highly viable across all cell densities (Figure S11, Supporting Information). Constructs with starting cell densities of 20, 50, and 100 M cells mL^−1^ displayed increasing cell retention, while those with 200 M cells mL^−1^ exhibited a significant reduction in cell retention. This reduction is likely due to the inability of the polymer network to sufficiently entrap cells at such a high density, resulting in the leakage of cells into the culture media within the first few hours. Consequently, constructs from the 20, 50, and 100 M cells mL^−1^ groups were further cultured for 3 days. As observed in Figure S12 (Supporting Information), constructs with 20 M cells mL^−1^ collapsed by D3, unable to maintain their integrity due to insufficient cell condensate formation following rapid hydrogel degradation, similar to the dead control samples. In contrast, constructs with 50 and 100 M cells mL^−1^ retained their structure, with the 50 M cells mL^−1^ constructs exhibiting greater deformation. However, the constructs with a cell density of 50 M cells mL^−1^ were too fragile to be moved out of the plate well, prompting us to determine that a density of 100 M is optimal for subsequent studies.

**Figure 6:**
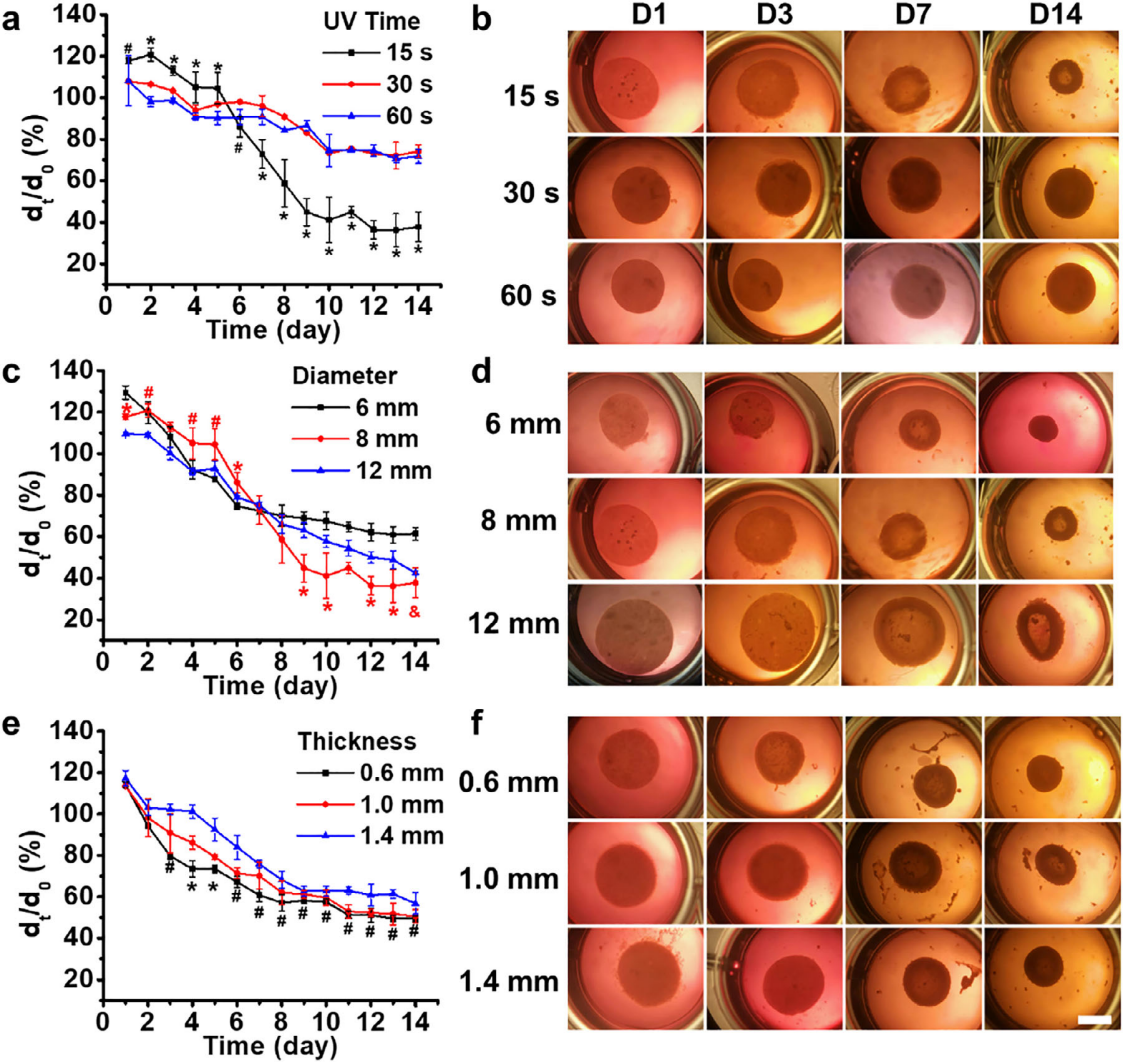
Impact of hydrogel stiffness and construct dimensions on morphing capacity. Effect ofa,b) hydrogel stiffness (photocrosslinking time), c,d) construct diameter, and e,f) construct thickness on construct morphing. For (a): ^*^ *p* < 0.05 compared to 30 and 60 s constructs, ^#^ *p* < 0.05 compared to 30 s constructs. For (c): ^*^ *p* < 0.05 compared to 6  and 12 mm constructs, ^#^ *p* < 0.05 compared to 12 mm construct, ^&^ *p* < 0.05 compared to 6 mm construct. For (e): ^*^ *p* < 0.05 compared to 1.0  and 1.4 mm constructs, ^#^ *p* < 0.05 compared to 1.4 mm construct. Scale bar: 5 mm. Unless otherwise specified, the preparation parameters for all constructs were as follows: cell density: 100 M cells mL^−1^; UV exposure: 15 s at 20 mW cm^−^^2^; disc dimensions: d_0_ = 8.0 mm, h = 1.0 mm.

To investigate the impact of hydrogel stiffness on construct morphing, constructs were prepared with varying photocrosslinking times, ranging from 15 to 60 s. Constructs subjected to longer photocrosslinking times exhibited increased rigidity. Consequently, cell‐laden constructs with 30 and 60 s photocrosslinking times showed minimal contraction and limited upward curving during the two‐week culture period (**Figure** **6**a,b). Interestingly, during the initial 5 days, constructs with a 15 s photocrosslinking time exhibited less contraction compared to those with 30 and 60 s photocrosslinking. This difference is attributed to the higher initial swelling of the 15 s constructs, resulting in greater volume expansion. After D5, CCFs induced significant contraction in the 15 s constructs, leading to a smaller construct size and more pronounced morphing compared to the 30 s and 60 s constructs.

Next, we examined the impact of construct dimensions, including diameter and thickness, on shape morphing (Figure 6c–f). All constructs underwent rapid contraction and shape morphing over 14 days of culture. However, among constructs of varying diameters, the 8 mm constructs exhibited greater contraction after D8 than the 6 and 12 mm constructs and maintained their bowl‐like shape by D14. In contrast, the 6 mm constructs formed cell clusters without a defined shape by D14 (Figure 6c,d). Rapid contraction and shape morphing across constructs with different thicknesses were also observed (Figure 6e,f). Among these, constructs with the smallest thickness of 0.6 mm displayed the most pronounced and rapid contraction and morphing. However, by D14, these constructs lost their shape, forming cell clusters. In comparison, 1.0 and 1.2 mm constructs maintained well‐defined bowl‐like shapes by D14.

The findings indicate that construct morphing is governed by a complex interplay of factors, including cell density, hydrogel stiffness, and construct dimensions. These parameters collectively offer a multifactorial strategy for programming the morphing capabilities of cell‐laden constructs. When designing CCF‐4D constructs, it is essential to strike a balance between the desired morphing potential and the ability to preserve a well‐defined shape over extended culture periods. This dual consideration is critical for achieving functional, stable tissue constructs in advanced 4D tissue engineering applications.

### 2.6 Impact of Initial Geometry on the Shape‐Morphing Capacity

**Figure 7:**
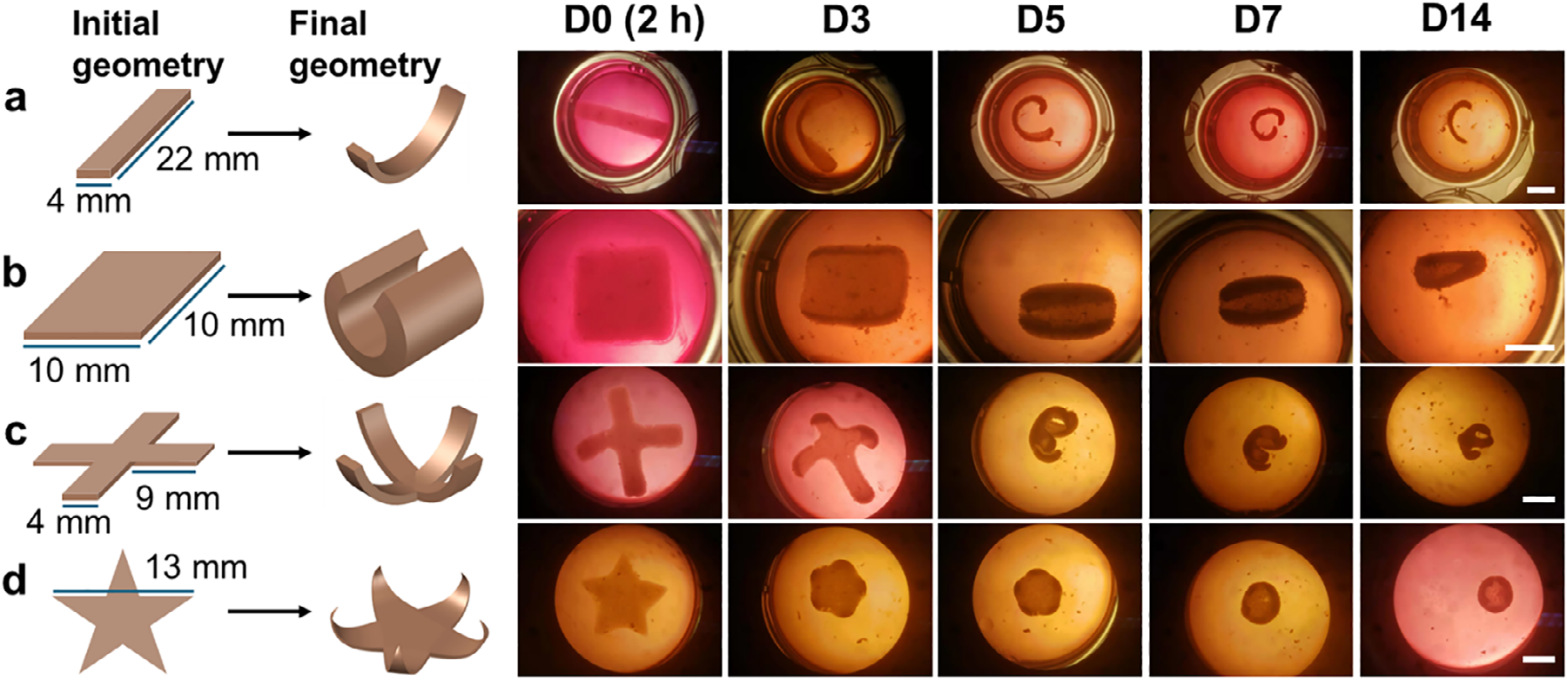
Shape morphing of cell‐laden constructs with varying initial geometries over 14 days of culture in media: a) strip‐shaped, b) square sheet‐shaped, c) four‐arm gripper‐shaped, and d) star‐shaped. Construct fabrication parameters: cell density: 100 M cells mL^−1^; UV exposure: 15 s at 20 mW cm^−^^2^. Construct thickness: 0.6 mm. Scale bars: 5 mm.

Given the demonstrated capability of CCF‐mediated shape morphing in disc‐shaped constructs, whether CCF could effectively drive shape changes to form different complex architectures was investigated by varying the initial geometric designs (**Figure** **7**). Consistent with the findings from disc‐shaped constructs, these constructs exhibited continuous and dynamic shape morphing throughout the culture period. Specifically, strip‐shaped constructs bent into a “C” pattern, reaching peak curvature at D7, followed by partial relaxation by D14 (Figure 7a). Similarly, sheet constructs rolled into tubular forms (Figure 7b), while the 4‐arm gripper geometry curled inward, resulting in a flower‐like configuration (Figure 7c). A comparable morphing process was observed in star‐shaped constructs (Figure 7d). Although printing patterns have been explored as a key parameter to modulate the shape‐morphing behavior of printed constructs,^[^ ^35^ ^]^ the different patterns investigated in this study, including grid, honeycomb, and gyroid, did not exhibit a noticeable impact on the shape transformation of the cell‐laden constructs (Figure S13, Supporting Information). This lack of distinction may be attributed to the printing conditions used, particularly the 60% infill density and a 22‐G needle, under which all filaments merged during printing, resulting in constructs with no discernible internal pattern. These results underscore the potential of leveraging initial geometric design to generate complex tissues through CCF‐driven shape morphing. This approach highlights the effectiveness of CCF in directing the programmed shape morphing of cell‐laden constructs, paving the way for biomimetic 4D engineering that replicates the dynamic morphogenesis observed in tissue development.

### 2.7 4D Tissue Engineering

In the human body, tissue development is inherently tied to morphological evolution, characterized by the formation of specific architectures through a dynamic morphing process that includes buckling, folding, elongation, curling, and/or wrinkling. This morphogenetic process is crucial in the development of complex tissue curvatures observed in structures such as intestinal villi and epithelial tubes.^[^ ^36^ ^]^ Not only does this process expand structural complexity to facilitate functionality, such as increasing surface area to volume ratio, as seen in branching morphogenesis, but it also provides biomechanical cues that are vital for guiding tissue development.^[^ ^37^, ^38^ ^]^ Therefore, introducing dynamic elements that mimic the morphological dynamics of native tissues may be valuable for advancing tissue engineering practices to a horizon of more effective tissue formation and to gain a deeper understanding of developmental processes.

Human mesenchymal stem cells (hMSCs), which exhibit fibroblast‐like morphology, have also been shown to induce significant contraction in hydrogel constructs.^[^ ^39^ ^]^ In our study, both hMSCs and NIH3T3 cells demonstrated comparable capacity to contract printed and photocrosslinked OMAGM hydrogel constructs. While constructs encapsulating NIH3T3 cells exhibited slightly greater contraction, the difference was not statistically significant (Figure S14, Supporting Information). To evaluate the ability of CCFs to drive shape morphing in engineered tissues, multipotent hMSCs were used to print cell‐laden constructs, which were then cultured in tissue‐specific environments to promote lineage‐specific tissue maturation.

**Figure 8:**
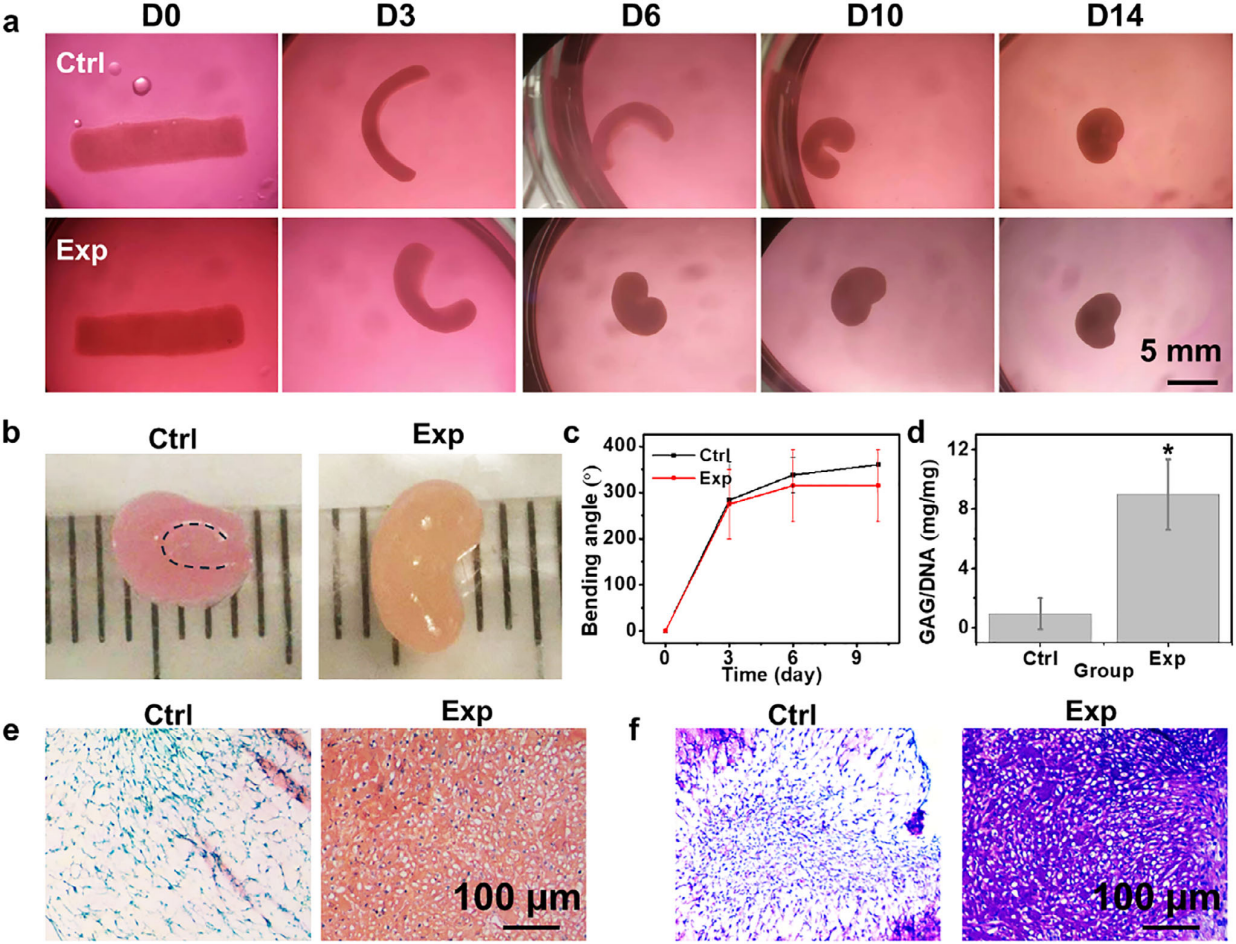
Differentiation study of 4D constructs for chondrogenesis. a) Images of constructs at different times during a 14‐day culture period. b) Images of D14 constructs removed from culture media, with each ruler tick mark representing 1 mm. The dotted line in the Ctrl sample outlines the curvature arc. c) Quantitative analysis of the bending angle change in Ctrl and Exp groups over time. d) Biochemical analysis of GAG production normalized to DNA content (GAG/DNA) in Ctrl and Exp constructs at D14. ^*^ *p *< 0.05. Histological staining of D14 constructs using e) SafO and f) TBO. Ctrl represents the control group cultured in growth medium, while Exp denotes the group cultured in chondrogenic differentiation medium.

Initially, hMSC chondrogenesis was induced within chondrogenic differentiation media to engineer cartilage‐like tissues using a simple strip‐shaped prototype (termed Exp group). A control group (Ctrl) was maintained in normal growth medium. Over the culture period, both Ctrl and Exp constructs exhibited continuous morphological evolution, with the Exp constructs demonstrating more rapid and pronounced volume shrinkage compared to the Ctrl group (**Figure** **8**a,b). However, no statistical significances were observed in the bending angle (Figure 8c). By D10, the Exp constructs had transformed from a “C” shape into a “kidney bean” shape, while the Ctrl constructs maintained their “C” geometry. The more pronounced geometrical changes in the Exp group may be attributed to the differentiated chondrocytes exerting stronger CCFs compared to the undifferentiated hMSCs in the Ctrl group.^[^ ^40^ ^]^ Biochemical assays revealed significantly higher glycosaminoglycan (GAG) production normalized to DNA content (GAG/DNA) in Exp constructs compared to Ctrl constructs (Figure 8d; Figure S15, Supporting Information), indicative of robust chondrogenesis driven by effective cell differentiation within the chondrogenic medium. These findings were corroborated by histological analyses using safranin O (SafO) and toluidine blue O (TBO) staining (Figure 8e,f).

**Figure 9:**
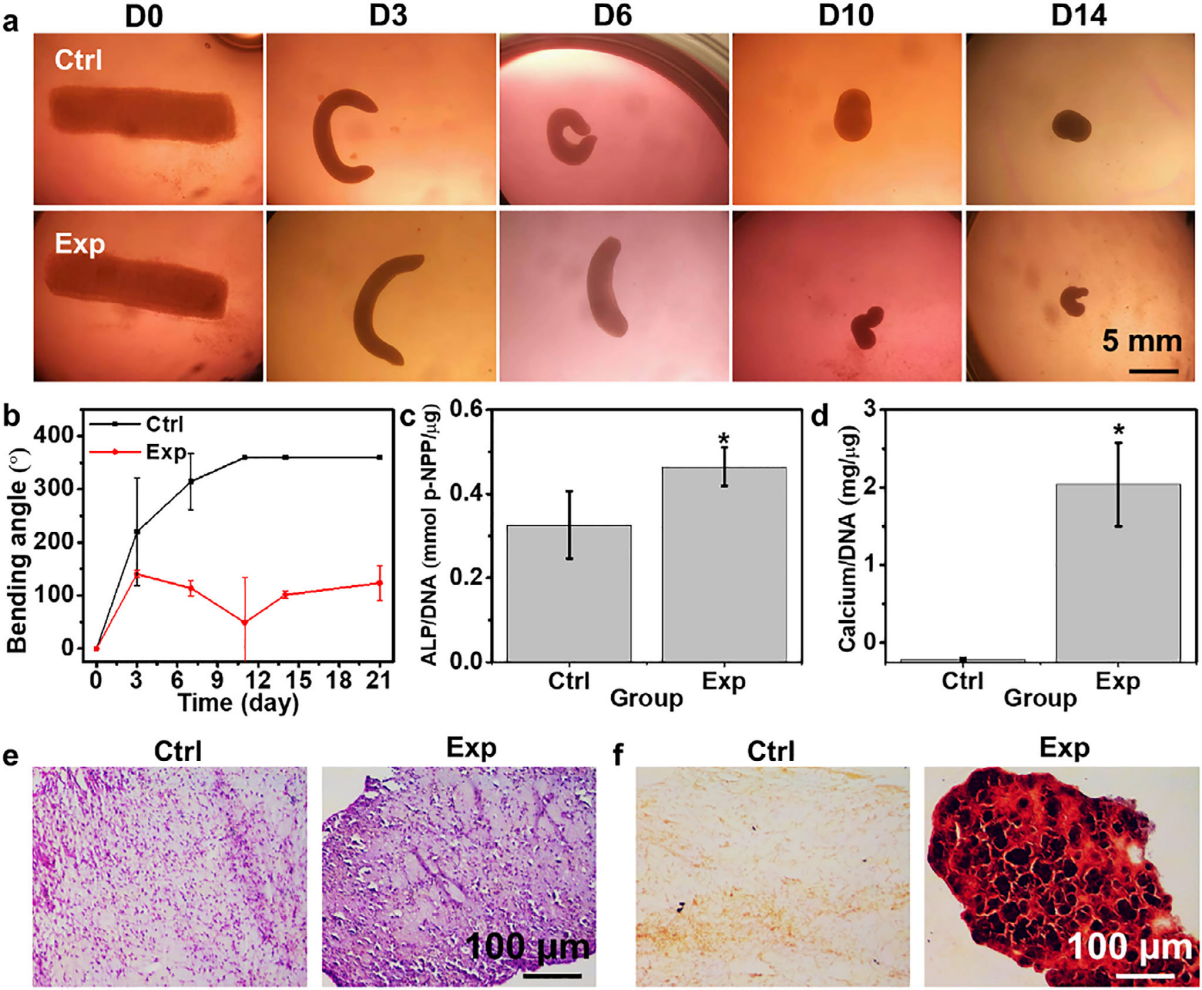
Differentiation study of 4D constructs for osteogenesis. a) Images of constructs at different times during a 14‐day culture period. b) Quantitative analysis of the change in bending angle for Ctrl and Exp groups over time. Quantification of c) ALP activity normalized to DNA (ALP/DNA) and d) calcium content normalized to DNA (Ca/DNA) in Ctrl and Exp constructs at D14. ^*^ *p* < 0.05. Histological staining of D21 constructs using e) H&E and f) ARS. Ctrl represents the control group cultured in growth medium, while Exp denotes the group cultured in osteogenic differentiation medium.

Osteogenic differentiation studies further validated the CCF‐4D engineering of bone tissues. Constructs cultured in media displayed progressive shape changes over a 21‐day period (**Figure** **9**a). They formed distinct “C” shapes by D6. However, the Ctrl constructs lost this geometric characteristic by D14, whereas the Exp constructs underwent significant shrinkage, retaining a small “C” shape. The Exp constructs, subjected to osteogenic differentiation, exhibited less pronounced bending angles compared to the Ctrl group throughout the culture period (Figure 9b), likely due to the production of mineralized ECM, which increased tissue rigidity and rapidly balanced the stress between cells and their surrounding matrix. This rigidity led to reduced tissue deformation in the Exp constructs, even though differentiated osteoblasts, known for their larger size, can exert higher traction forces compared to undifferentiated stem cells.^[^ ^41^ ^]^ Biochemical analysis at D21 confirmed the differentiation capabilities within the osteogenic media, as evidenced by significantly elevated levels of alkaline phosphatase (ALP) and calcium (Figure S16, Supporting Information), both normalized to DNA content (ALP/DNA and Ca/DNA, respectively), in the Exp group relative to the Ctrl group (Figure 9c,d). Histological assessments using H&E and alizarin red S (ARS) staining further demonstrated denser tissue organization and more intense red staining in the Exp constructs, indicative of neo‐bone‐like tissue formation (Figure 9e,f).

Collectively, these chondrogenic and osteogenic studies highlight the ability of the 4D constructs to support differentiation while undergoing geometric transformations, thereby affirming their potential as models for tissue development. Throughout tissue maturation, cells within the transient hydrogel matrix actively remodel the ECM, ultimately forming complex tissues in the absence of hydrogel materials. This dynamic process closely mimics the native tissue development observed in vivo, underscoring the value of coupling 4D morphogenesis with tissue engineering to better replicate the intricacies of in vivo tissue formation.

Compared to conventional 4D systems that primarily rely on physical and chemical stimuli to elicit shape morphing, leveraging biological forces, such as CCFs generated by live cells, represents a significant advancement in biomimetic 4D tissue engineering. However, due to the inherently weak nature of CCFs, existing CCF‐driven 4D systems often require ultrasoft hydrogel matrices to enable detectable shape transformation.^[^ ^19^, ^20^, ^42^ ^]^ While these soft hydrogels facilitate cellular force transmission, they pose challenges in forming stable, freestanding structures, necessitating the use of mechanical support to maintain the integrity of the initial cell‐laden constructs. To overcome this limitation, our recent work on CCF‐driven 4D systems introduced a mechanically self‐adaptive hydrogel scaffold incorporating sacrificial gelatin microspheres.^[^ ^40b^ ^]^ This design allows for the fabrication of stable 3D freestanding structures that undergo controlled softening during culture as the gelatin microspheres dissolve. This progressive softening amplifies the impact of CCFs, enabling significant shape transformations over time. While hydrogel materials play a critical role in maintaining structural integrity during culture and differentiation, their prolonged presence could interfere with tissue remodeling and introduce potential immunotoxicity when applied in vivo. To address these challenges, we have developed a mechanically robust yet rapidly degradable hydrogel system that supports stable 3D printing while facilitating the formation of cell condensates capable of programmed 4D shape morphing. Upon culture, this system enables the formation of complex cell condensate architectures devoid of residual hydrogel biomaterials, offering a CCF‐based dynamic platform that closely mimics the morphogenesis of native tissue development. However, in this system, the morphing directionality is primarily dictated by the spatial restriction imposed by the plate bottom, which lacks a mechanism to precisely control morphing directions, thereby limiting the range of achievable morphologies. Incorporating signaling molecules or spatially aligning cell positions could provide a means to regulate CCF directionality in future studies, enabling more precise and programmable shape transformations to better emulate complex morphogenetic processes.

## 3 Conclusion

This work presents an innovative 4D bioprinting platform that uniquely utilizes CCF as the sole stimulus to orchestrate shape transformation. The bioink, composed of degradable OMA microgels integrated with cell‐adhesive GelMA, was formulated to ensure smooth cell loading and printing. The engineered hydrogel scaffolds served as temporary mechanical supports, maintaining structural integrity while rapidly degrading in synchrony with morphogenesis during culture. This process facilitated the generation of tissue constructs with complex, deformed architectures, devoid of residual polymeric materials. By precisely programming CCF within geometrically defined constructs, global shape transformation in large constructs was achieved, resulting in the formation of tissues with complex architectures. Utilizing this unique CCF‐driven 4D bioprinting platform, 4D morphogenesis through proof‐of‐concept studies in cartilage and bone tissue engineering was demonstrated. This CCF‐4D bioprinting approach represents a significant advancement in regenerative medicine, with broad potential applications in biomimetic tissue engineering and beyond.

## 4 Experimental Section

### OMA Microgel (OMA MG) Synthesis

OMA was synthesized according to previously published protocols.^[^ ^21a^ ^]^ Briefly, 10 g of alginate was dissolved in 900 mL of deionized Milli‐Q water overnight. Next, 0.545 g sodium periodate solution in 100 mL of deionized water was added to the alginate solution. The mixture was kept in the dark for 24 h under stirring at room temperature. Subsequently, 19.52 g of 2‐(N‐morpholino)ethanesulfonic acid (MES, Sigma, Cat#M8250‐1KG) and 17.53 g of sodium chloride were added, and the pH was adjusted to 6.5 using 5N sodium hydroxide. Then, 0.589 g of N‐hydroxysuccinimide (NHS, VWR, cat#102614‐812) and 7.776 g of 1‐ethyl‐3‐(3‐dimethylaminopropyl)carbodiimide hydrochloride (EDC∙HCl, Oakwood, cat#024810‐250g) were added under vigorous stirring. After 10 min, 0.844 g of 2‐aminoethylmethacrylamide hydrochloride (AEMA, PolySciences, cat#21002‐10) was then added, and the solution was left to react for 24 h under stirring in the dark. After precipitation using chilled acetone, the alginate was rehydrated and dialyzed against deionized Milli‐Q water for the next 3 days using MWCO 3500 Da dialysis tubing (Spectrum Laboratories Inc., cat#086705B). The product was treated with charcoal (Neta Scientifics, cat#099536) for 30 min and then lyophilized for 14 days. The structure of OMA was confirmed using ^1^H NMR analysis on the Bruker 600 MHz AVANCE III NMR Spectrometer and shown in Figure S1 (Supporting Information). The actual methacrylation was determined to be 9.1%.

The OMA polymer (2 g) was dissolved in deionized water (100 mL) at 2% w/v solution and then crosslinked in a 0.2 m CaCl_2_ solution (1 L) for 12 h. Next, the OMA hydrogel was collected and fragmented finely in a blender (Osterizer MFG, at “pulse” speed) for 5 min in the presence of 70% ethanol (EtOH, 100 mL). The mixture was then centrifuged and stored in 70% EtOH at −20 °C for future use.

### GelMA Synthesis

GelMA was synthesized according to previously published protocols.^[^ ^43^ ^]^ Briefly, 20 g of Gelatin Type B (Sigma, cat#G9391‐100G) was dissolved in PBS (pH 7.4). Then, 10 mL of methacrylic anhydride (Sigma, cat#276685‐500ML) was added to the solution slowly (1 mL min^−1^) while stirring vigorously. The reaction was maintained for 1 h at 50 °C with stirring, and then at room temperature overnight at a pH of 9. The next day, GelMA was precipitated in excess acetone at 4 °C, and then purified by dialysis (MWCO 12–14 kDa, Spectrum Laboratories Inc., cat#132709T) against deionized Milli‐Q water for 10 days at 50 °C. The final product was obtained by lyophilization for 10 days.

### Cell Culture

NIH3T3 fibroblasts (ATCC) were expanded in growth media composed of Dulbecco's Modified Eagle Medium‐low glucose (DMEM‐LG, Sigma, cat#D5523‐50L) with 10% fetal bovine serum (FBS, Sigma, cat#F19) and 1% penicillin/streptomycin (P/S, Gibco, cat#15140122) until the cells reached 85% confluency. The medium was changed every other day. On the day of printing, the cells were harvested and suspended at 1 × 10^8^ cells mL^−1^ composite bioink. After printing, the constructs were cultured in Dulbecco's Modified Eagle Medium‐high glucose (DMEM‐HG, Sigma, cat#D7777‐10×1L) supplemented with 10% FBS and 1% P/S.

### Cell‐Laden Composite Bioink Preparation

On the day of printing, the OMA MGs stored in 70% EtOH were washed four times with deionized water containing 0.05% w/w photoinitiator (2‐hydroxy‐4′‐(2‐hydroxyethoxy)‐2‐methylpropiophenone, Sigma, cat#410896‐10g), followed by a wash with DMEM‐HG containing 0.05% w/w photoinitiator. Gas chromatography confirmed the absence of ethanol in the resulting OMA slurry, ensuring its suitability for subsequent applications. Lyophilized GelMA was then added to the recovered OMA MGs at a concentration of 3% w/v and vortexed vigorously to yield OMAGM bioinks. Next, cells were added to the OMAGM at a concentration of 100 M cells mL^−1^ bioink. The cell‐laden bioink in a syringe was mounted on a BIOX 3D printer (Cellink, San Diego, CA) and was then printed using the parameters listed in Table S1 (Supporting Information) file. After printing, the constructs were further UV‐crosslinked (20 mW cm^−2^) for a specific duration. The constructs were then transferred into culture media and placed in an incubator at a constant temperature of 37 °C for further investigation. Disc‐shaped constructs were cultured in 24‐well plates with 2 mL of media, while strip, square, gripper, and star‐shaped constructs were cultured in 12‐well plates with 4 mL of media. Differentiation strip constructs were cultured in 6‐well plates with 8 mL of media. In all cultures, half of the media volume was changed daily.

### 4D Printed Chondrogenesis Constructs

In this study, hMSCs were isolated and harvested according to previously established protocols.^[^ ^44^ ^]^ hMSCs at passage 4 were mixed with OMAGM to generate hMSCs‐laden bioinks (100 M cells mL^−^ bioink), which were then printed into constructs and crosslinked as described above. The constructs were then cultured in chondrogenic medium composed of DMEM‐HG (Sigma, cat#D5648‐10 × 1L), 1% v/v P/S, 10% v/v ITS^+^ (BD Biosciences, cat#354352), 1% v/v nonessential amino acids (NEAA, Gibco, cat#11140050), 100 mm sodium pyruvate (Fisher, cat#SH3023901), 10^−7^ m dexamethasone (Sigma, cat#D4902‐100mg), 129 nm L‐ascorbic acid phosphate (Wako USA, cat#013‐12061), and 10 ng mL^−1^ transforming growth factor beta‐1 (TGF‐β1, Peprotech, cat#100‐21‐10UG). The culture medium was changed every day. A control group was cultured in normal growth medium (DMEM‐HG supplemented with 10% FBS and 1% P/S) for comparison. The constructs were cultured for 14 days until collection. Sample sizes of *N = 4* were collected for biochemical analysis and *N = 2* were collected for histological staining.

### 4D Printed Osteogenesis Constructs

The hMSCs at passage 3 (100 M cells mL^−1^ bioink) were used to prepare the cell‐laden OMAGM bioinks for printing. The printed constructs were cultured in differentiation media composed of DMEM‐HG, 100 ng mL^−1^ bone morphogenetic protein‐2 (BMP‐2, Genscript, cat#Z02913), 10% v/v FBS, 1% v/v P/S, 100 µm dexamethasone, and 100 µm L‐ascorbic acid phosphate. Constructs in the control group were cultured in normal growth media for comparison. The constructs were cultured for 14 days until collection. Sample sizes of *N = 4* were collected for biochemical analysis and *N = 2* were collected for histological staining.

### Histology

Staining was performed according to previously established protocols.^[^ ^45^ ^]^ Briefly, samples were submerged in 10% neutral buffered formalin at 4 °C overnight. The samples were then dehydrated, paraffin‐embedded, and sectioned at 7 µm thickness using a Leica RM2255 microtome (Leica Biosystems, Nussloch, Germany). Next, the samples were stained with Mayer S hematoxylin (Fisher, cat#TA125MH) and eosin (Electron Microscopy Science, cat#26763‐03) (H&E), phalloidin (Fisher, cat #a12380), and E‐cadherin (Fisher, cat#560061) to observe cell morphology and tissue organization. To examine GAG production, a combination of 0.1% w/v SafO (Acros Organics, cat#AC419210250) and counterstain of 1% w/v fast green (Fisher, cat#F99‐10), or TBO (Fisher, cat#BP107‐10) staining was used.^[^ ^45a^ ^]^ Briefly, SafO was applied for 5 min, followed by 2 min of fast green. TBO, at pH 4.0, was applied for 30 min on the samples. Additionally, ARS (Sigma, cat#A5533‐25G) staining was used to examine calcium mineralization deposits according to a previously reported protocol.^[^ ^46^ ^]^

### Live/Dead Cell Staining

Live/dead cell staining was performed at predetermined time points during culture to assess cell viability using a previously established protocol.^[^ ^47^ ^]^ Briefly, constructs were stained with 16 µL of fluorescein diacetate (2 mg mL^−1^, Sigma, cat#F7378) and 8 µL of propidium iodide (2 mg mL^−1^, Sigma, cat#P4170‐25MG), respectively for 3 min, and then imaged on a Nikon Eclipse TE300 fluorescence microscope (Nikon, Tokyo, Japan) equipped with an AmScope MU1403 camera (AmScope, Irvine, California). *N = 3*.

### Biochemical Assays

DNA, GAG, ALP, and calcium contents were quantified according to the modified protocols from previous literature.^[^ ^45b^ ^]^ Briefly, prior to quantification, chondrogenesis samples were digested in 0.5 mL of a solution containing 50 µg mL^−1^ papain (papaya latex, Sigma, cat#3P4762), 2 mm L‐cysteine (Sigma, cat#C7352), 50 mm sodium phosphate (Sigma, cat#BP332‐500), and 2 mm EDTA (Fisher, cat# BP120‐1, pH 6.5) for 12 h at 65 °C. Next, 0.5 mL of nuclease‐free water was added to each sample, and the solution was vortexed for 1 min. DNA concentration was determined using a PicoGreen (Invitrogen, cat#P7581) dsDNA fluorescence assay on a plate reader (Molecular Devices ID5) with excitation at 480 nm and emission at 520 nm. GAG content was determined using a 1,9‐dimethylmethylene blue (Sigma, cat#341088) absorbance assay at 595 nm on the plate reader. *N = 4*.

Prior to quantification, osteogenesis samples were homogenized in 1 mL of Cellytic Buffer M (Sigma, cat#C2978‐250ML) for 90 s on ice. Next, the homogenates were centrifuged at 500× g for 5 min. DNA content was determined using the same assay as described for chondrogenesis samples. ALP content was quantified using an absorbance assay at 405 nm on the plate reader. Calcium content was determined using a calcium assay solution kit (Pointe Scientific, cat#C7503‐480) with absorbance measured at 570 nm on the plate reader. *N = 4*.

### Bending Angle Calculations

The bending angle was quantified according to a previously published protocol.^[^ ^8b^ ^]^ Briefly, the hydrogel bar was extended into a circle with equal crosshatches that divide the circle into four quadrants. The hydrogel bar with the circle and crosshatches was then loaded into NIH ImageJ for angle measurement. If the hydrogel bar curved past the half of the circle, the resulting bending angle was subtracted from 360° to obtain the bending angle. *N = 3*.

### Statistical Analysis

All data are reported as mean ± standard deviation (±SD). Statistical analysis was performed, and significance was determined using one‐way ANOVA with a post‐hoc Tukey HSD test. *p* < 0.05 was considered significant unless otherwise specified.

## Conflict of Interest

The authors declare no conflict of interest.

## Supporting information



Supporting Information

## Data Availability

The data that support the findings of this study are available from the corresponding author upon reasonable request.
