# Supplementary material for: Cell Contractile Force‐Mediated Morphogenetic Tissue Engineering via 4D Printed Degradable Hydrogel Scaffolds
Source: Adv Sci (Weinh). 2025 Sep 26;12(48):e07288. doi: 10.1002/advs.202507288 (PMC12752645; doi:10.1002/advs.202507288)
Supplement: Supplementary file 1 — Supporting Information [file ADVS-12-e07288-s001.docx]

Supporting Information

Cell Contractile Force-Mediated Morphogenetic Tissue Engineering via 4D Printed Degradable Hydrogel Scaffolds

Aixiang Ding, Kaelyn L. Gasvoda, David S. Cleveland, Sriramya Ayyagari, Eben Alsberg*


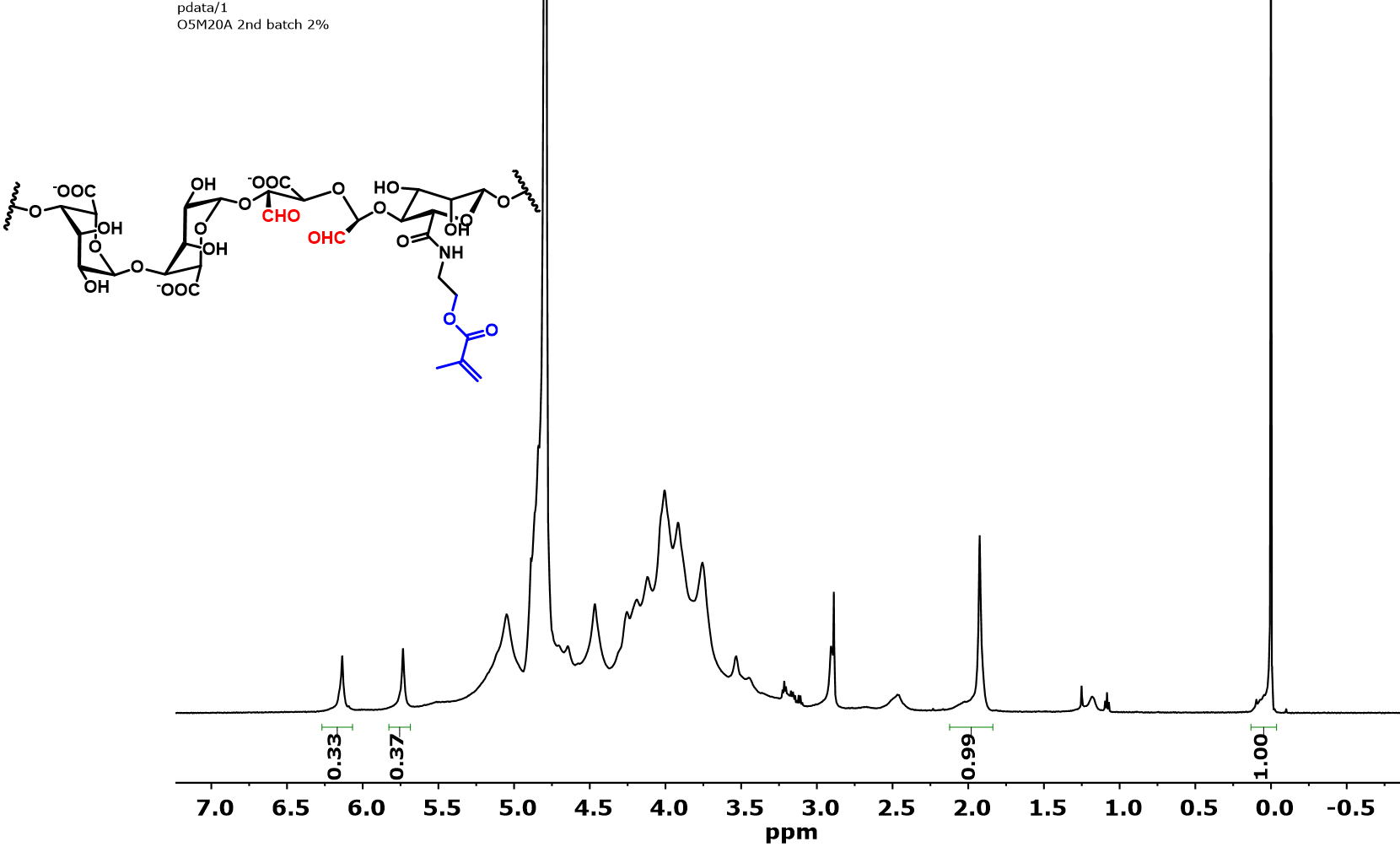


**Figure S1**. Molecular structure and ^1^H NMR (D_2_O, 500 MHz) of OMA.


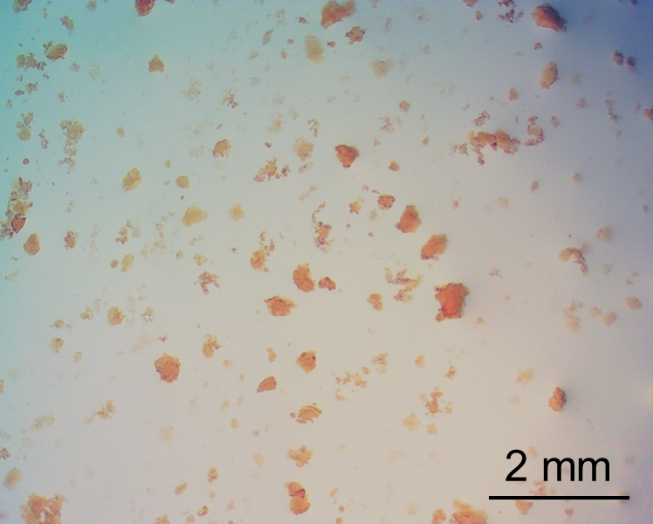


**Figure S2**. Photomicrograph of SafO-stained OMA microgels.


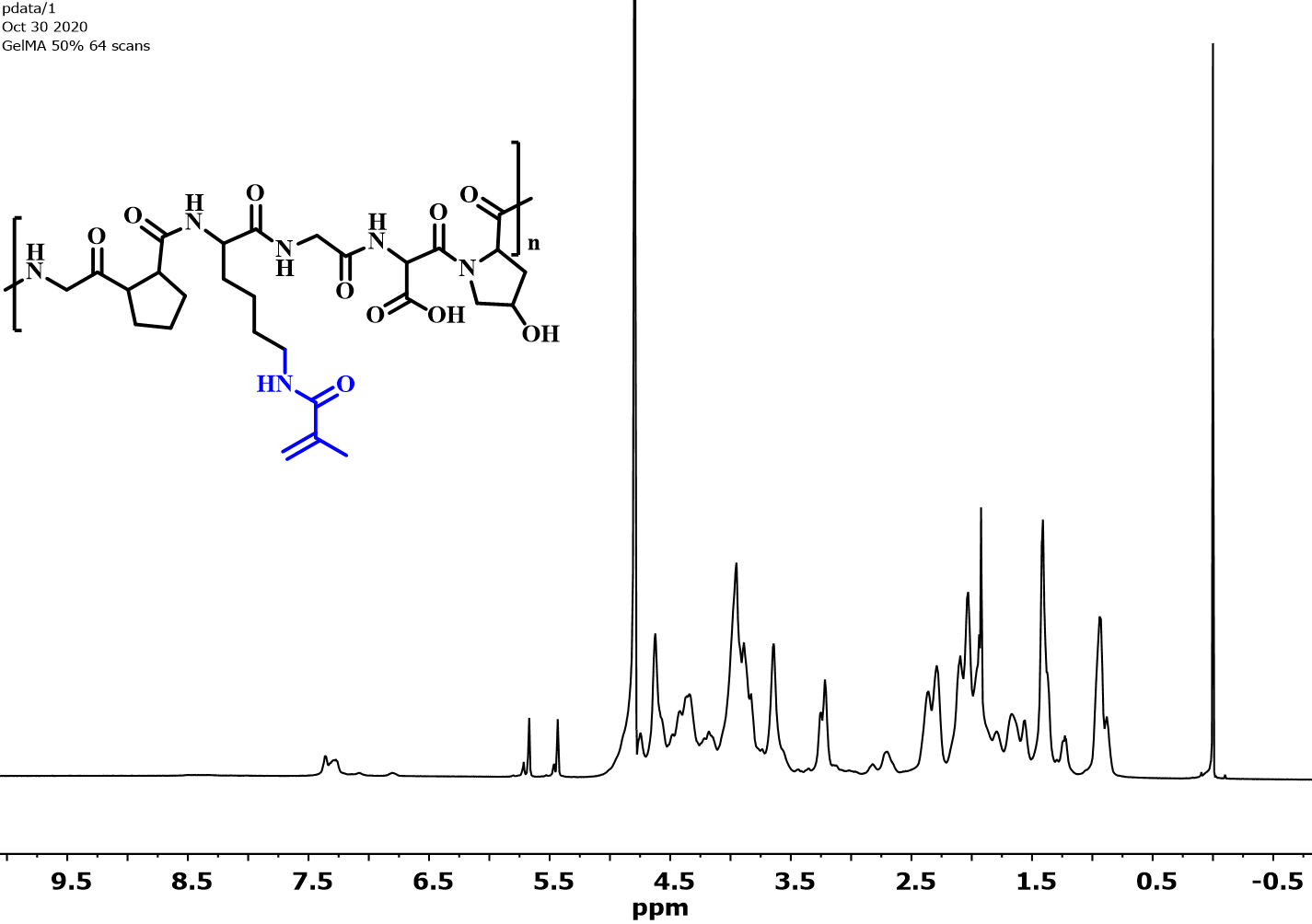


**Figure S3**. Molecular structure and ^1^H NMR (D_2_O, 500 MHz) of GelMA.


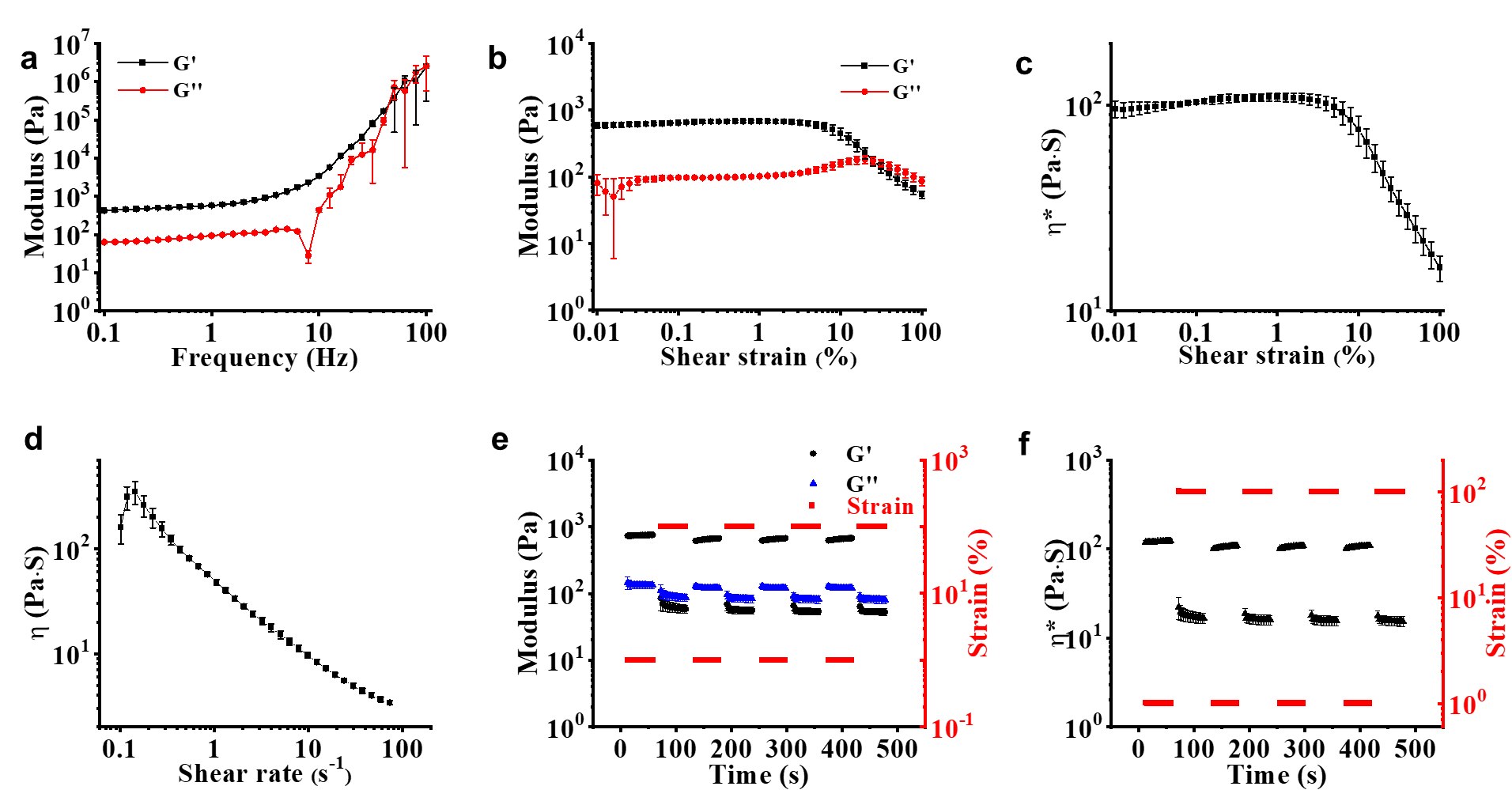


**Figure S4**. **Rheological properties of the OMA bioink.** (a) Storage modulus (G') and loss modulus (G'') as a function of frequency. (b) Changes in G' and G'' with increasing shear strain. (c) Complex viscosity (η*) as a function of shear strain. (d) Viscosity (η) as a function of shear rate. (e) Modulus and (f) complex viscosity changes over time under cyclic shear strain of 1% and 100%.

**Table S1**. 3D printing parameters.

| **Needle Size** | 22 G |
| --- | --- |
| **Printing Speed** | 4 mm/s |
| **Extrusion rate** | 1.2 μL/s |
| **Infill Density** | 60% |
| **Layer Height** | 0.66mm |
| **Printing Pattern** | Rectilinear |


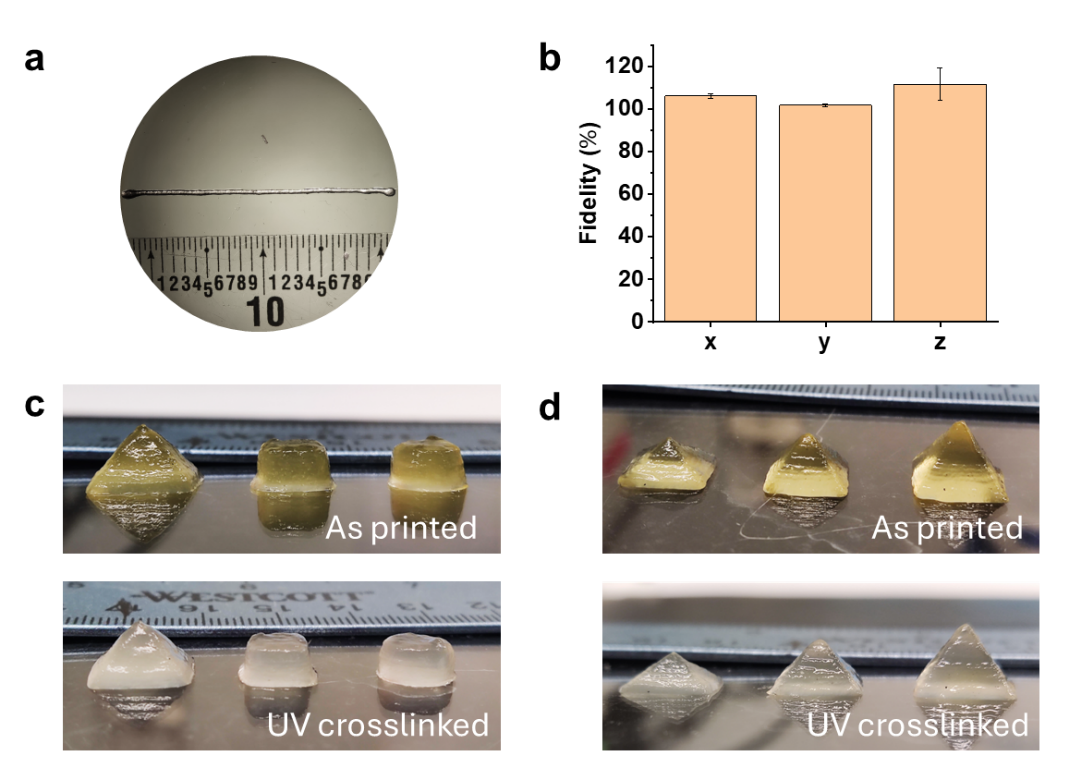


**Figure S5**. Printability characterization of OMAGM bioinks. (a) Optical image of a filament extruded through a 22-gauge needle (ID: 413 µm). (b) Printing fidelity assessment (%) along the x, y, and z axes. (c, d) Representative printed constructs: (c) various geometries including a pyramid (10 mm base × 6 mm height), cube (6 mm × 6 mm × 4 mm), and cylinder (8 mm diameter × 4 mm height), and (d) hydrogel pyramids of varying heights (from left to right: 10 mm × 4 mm, 10 mm × 6 mm, and 10 mm × 8 mm).


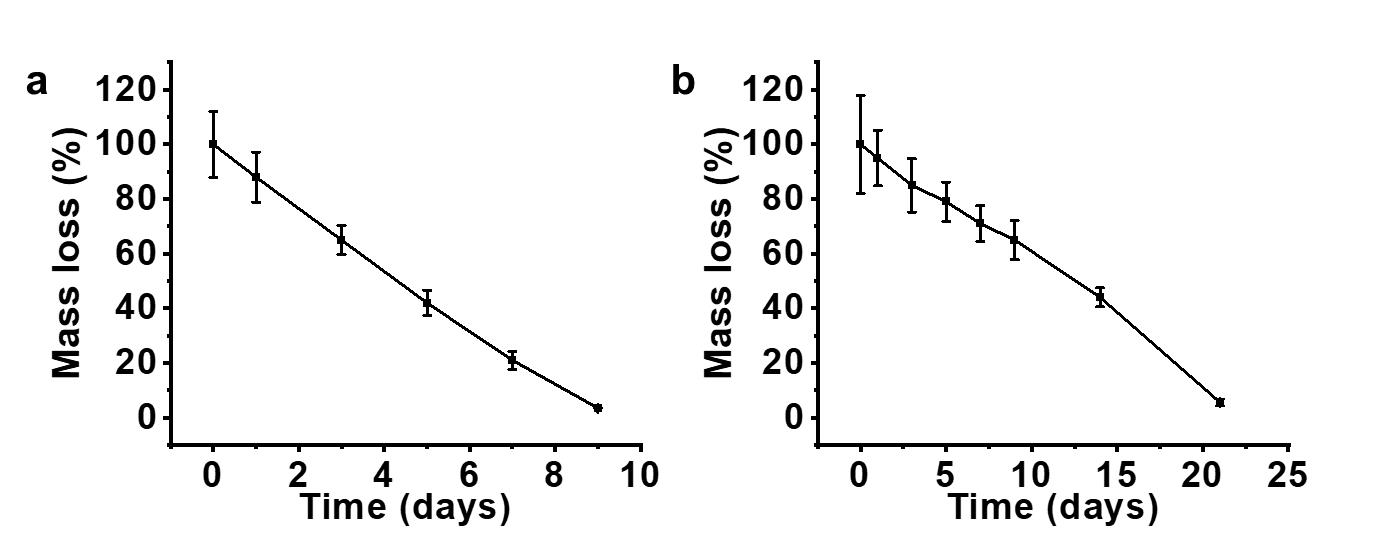


**Figure S6**. Mass loss profile of OMAGM hydrogels with UV crosslinking times of (a) 30 s and (b) 60 s.


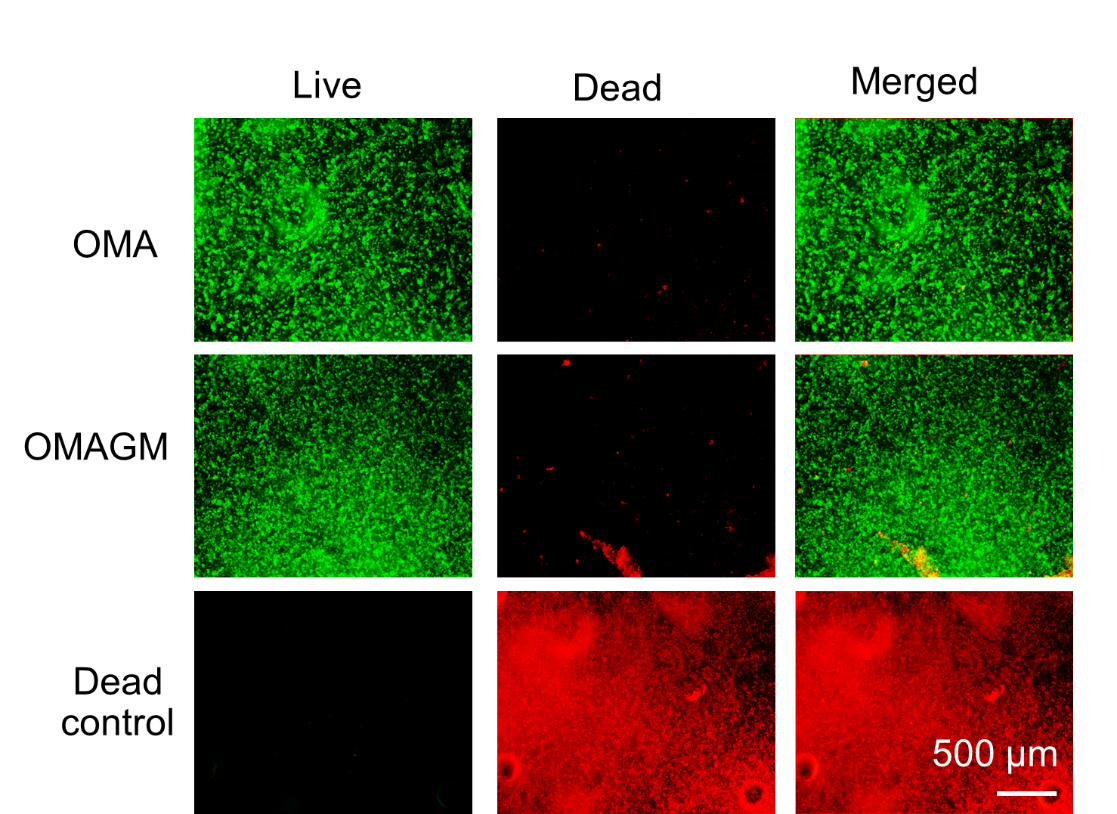


**Figure S7**. Live/dead staining images of cell-laden constructs following 4 hours of culture in cell growth medium. Conditions: cell density: 100M; UV: 15 s at 20 mW/cm^2^; disc dimensions: d_0_ = 8.0 mm, h = 1.0 mm.


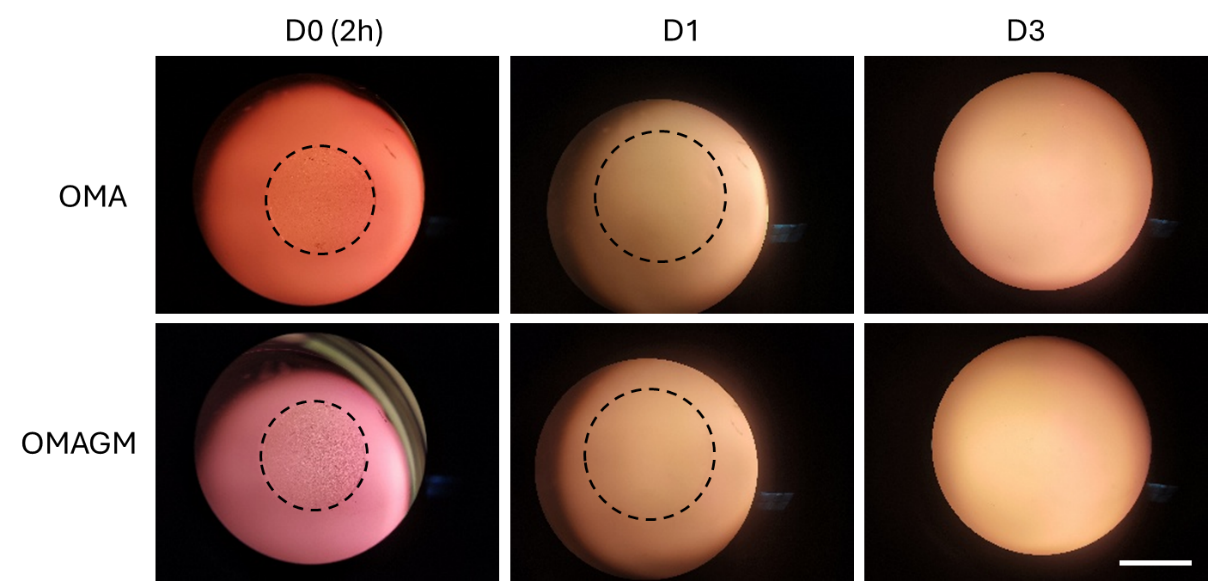


**Figure S8**. Photomicrographs of cell-free OMA and OMAGM hydrogel constructs at D0 (2 h), D1, and D3. Images at D0 (2 h) were acquired after 2 h of incubation in culture medium. By D3, the constructs collapsed due to extensive hydrogel degradation, and no images were collected thereafter. Scale bar = 5 mm. UV: 15s at 20 mW/cm^2^; disc dimension: d_0_ = 8.0 mm, h = 1.0 mm.


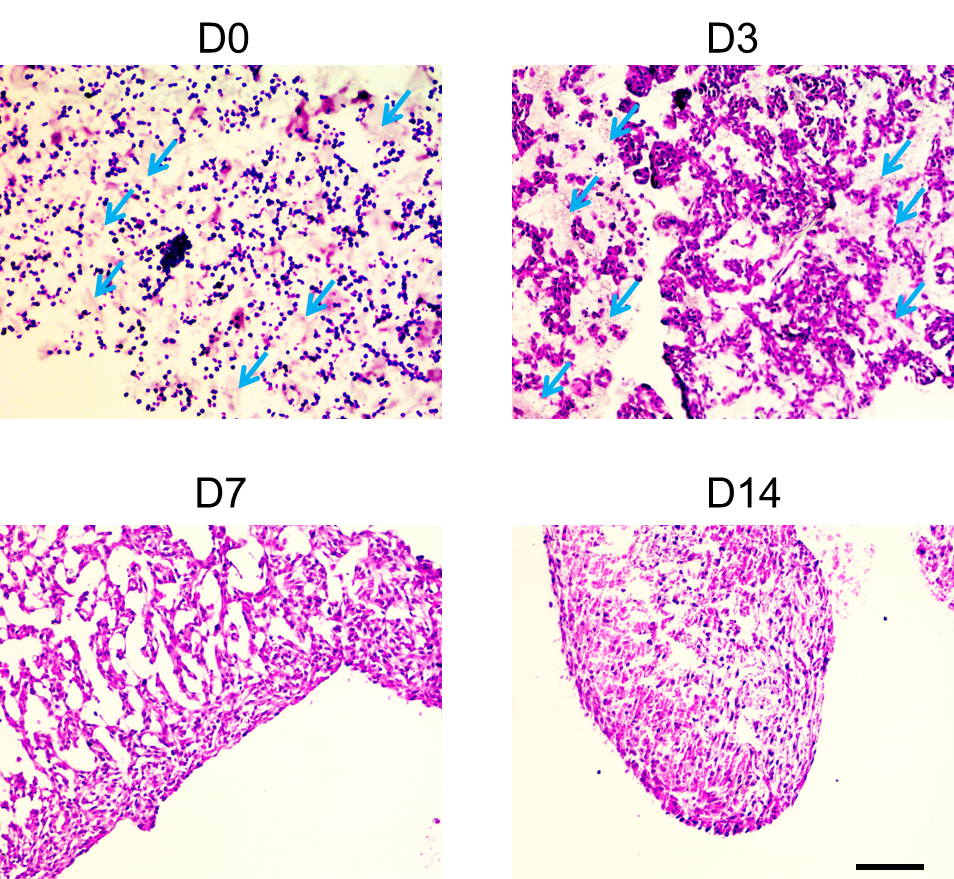


**Figure S9**. Representative H&E staining images of tissue-only constructs formed from the OMAGM group at different time points. Blue arrows in the D0 and D3 images highlight a few regions of the hydrogel matrix stained in light purple color. Scale bar: 100 μm.


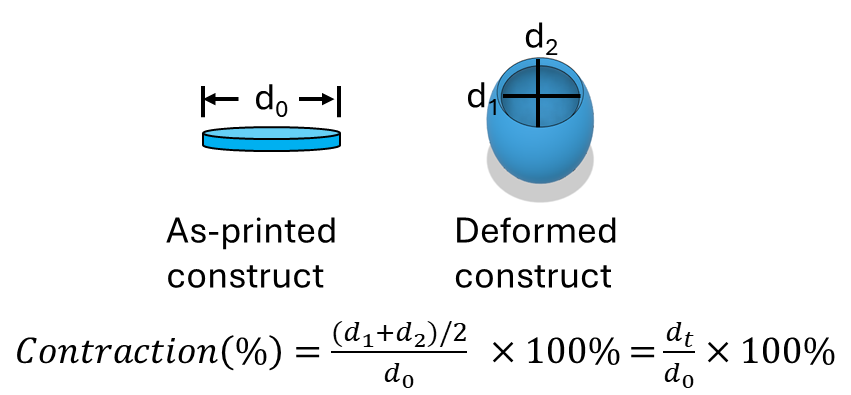


**Figure S10**. Quantification methodology of contraction deformation in cell-laden constructs.


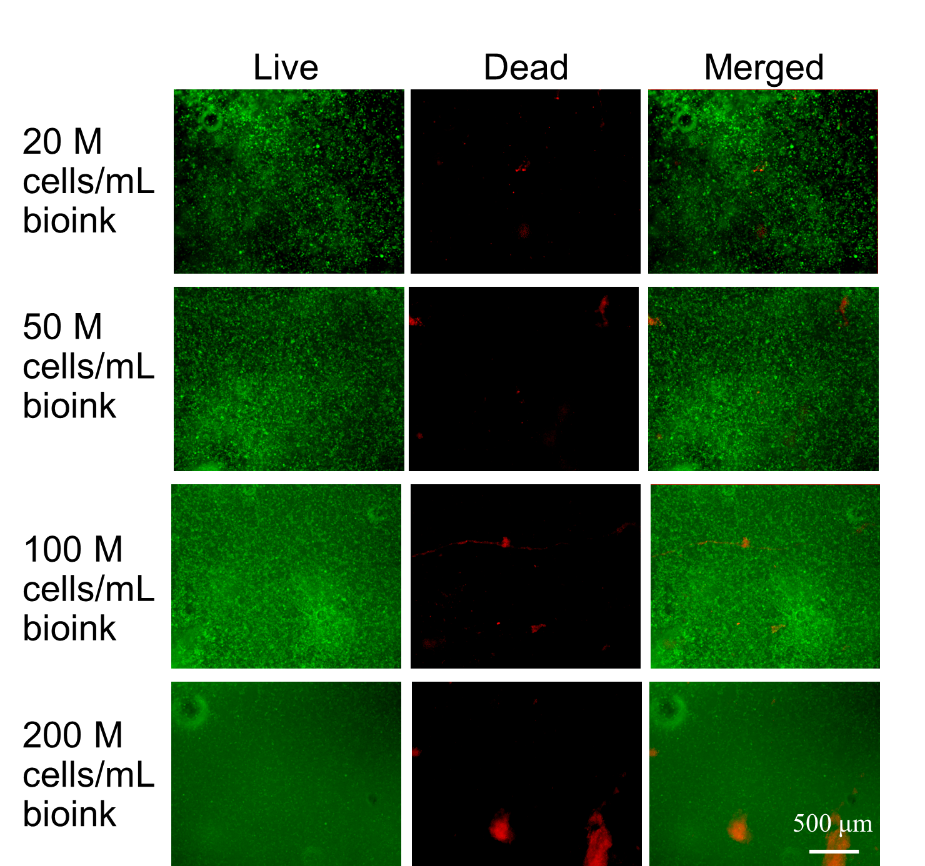


**Figure S11**. Live/dead staining images of cell-laden constructs at varying cell densities. Conditions: UV: 15 s at 20 mW/cm^2^; disc dimensions: d_0_ = 8.0 mm, h = 1.0 mm.


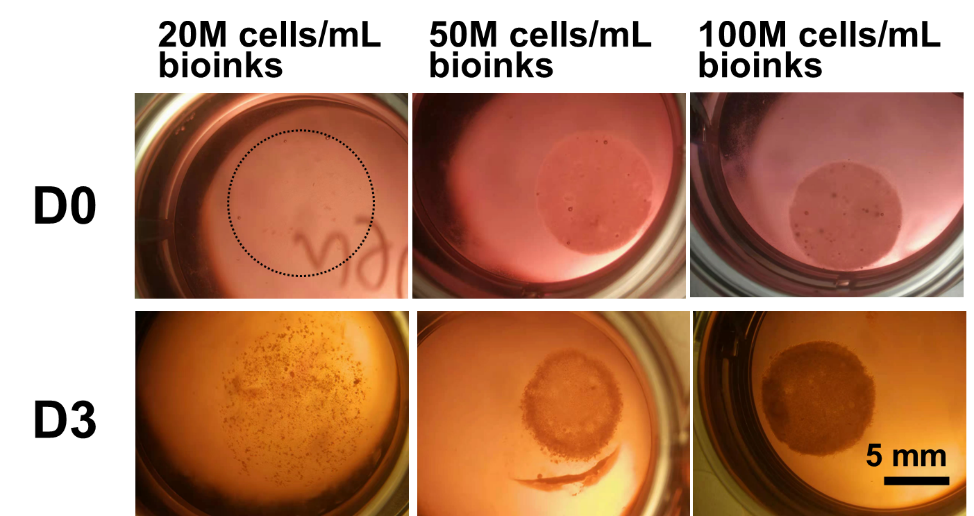


**Figure S12**. Representative images of disc constructs with varying cell densities at D0 and D3 of culture. Conditions: UV: 15 s at 20 mW/cm^2^; disc dimensions: d_0_ = 8.0 mm, h = 1.0 mm.


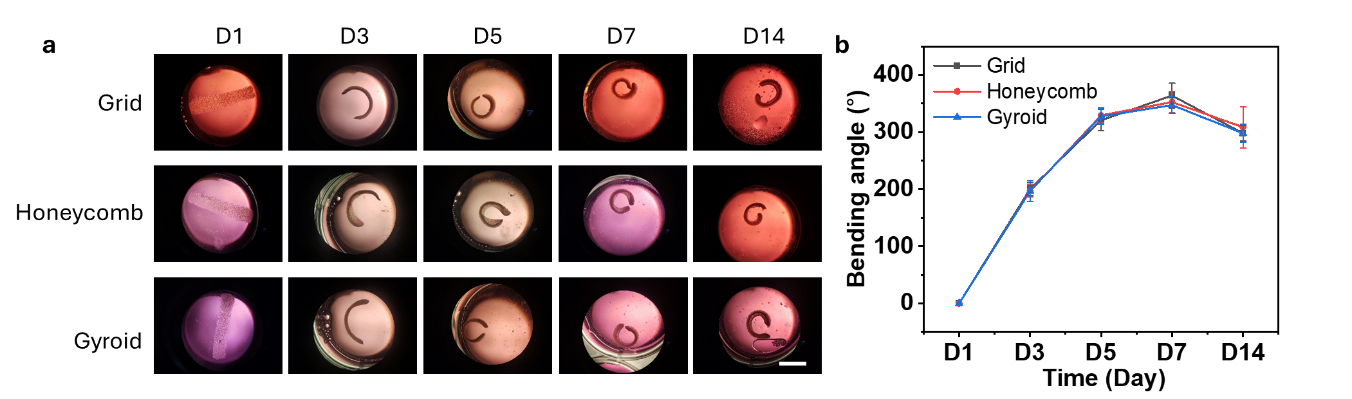


**Figure S13.** (a) Bending deformation and (b) angles of cell-laden strip-shaped constructs over the culture period. Construct fabrication parameters: cell density = 100M; UV exposure = 15 s at 20 mW/cm². Construct dimensions: 4 mm × 22 mm × 0.6 mm. Scale bar = 5 mm.


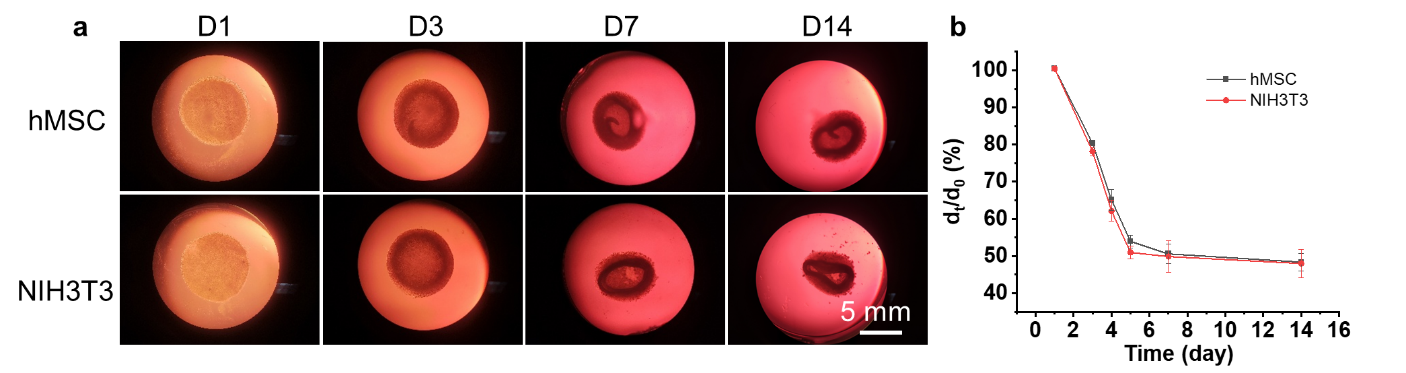


**Figure S14**. (a) Time-dependent shape changes of hydrogel constructs laden with hMSCs or NIH3T3 cells during culture. (b) Change in diameter ratio (d_t_/d_0_) over culture time. Construct fabrication parameters: cell density: 100M; UV: 15s at 20 mW/cm^2^; disc dimensions: d_0_ = 8.0 mm, h = 1.0 mm.


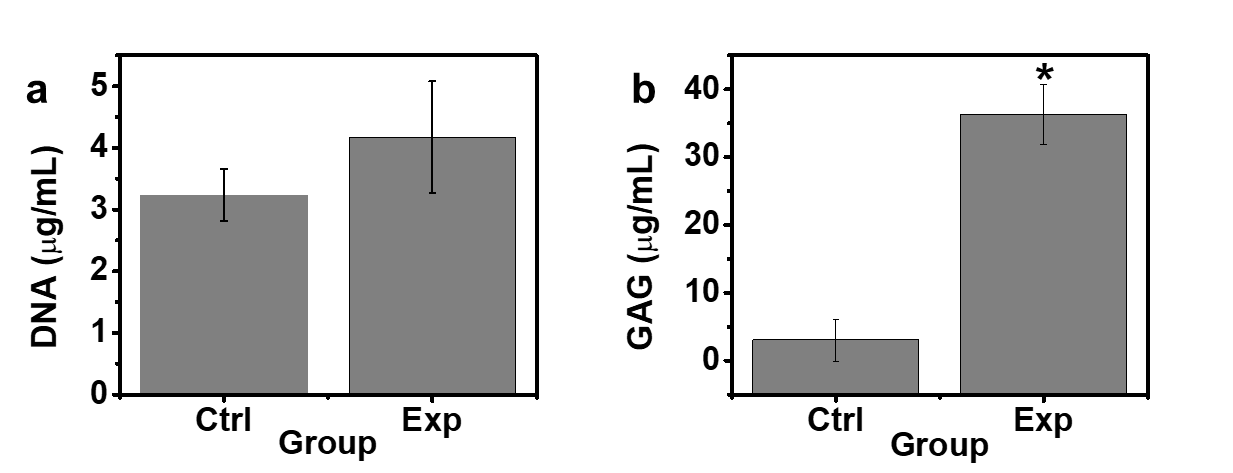


**Figure S15**. Biochemical analysis of (a) DNA and (b) GAG contents in Ctrl and Exp constructs at D14. *p < 0.05.


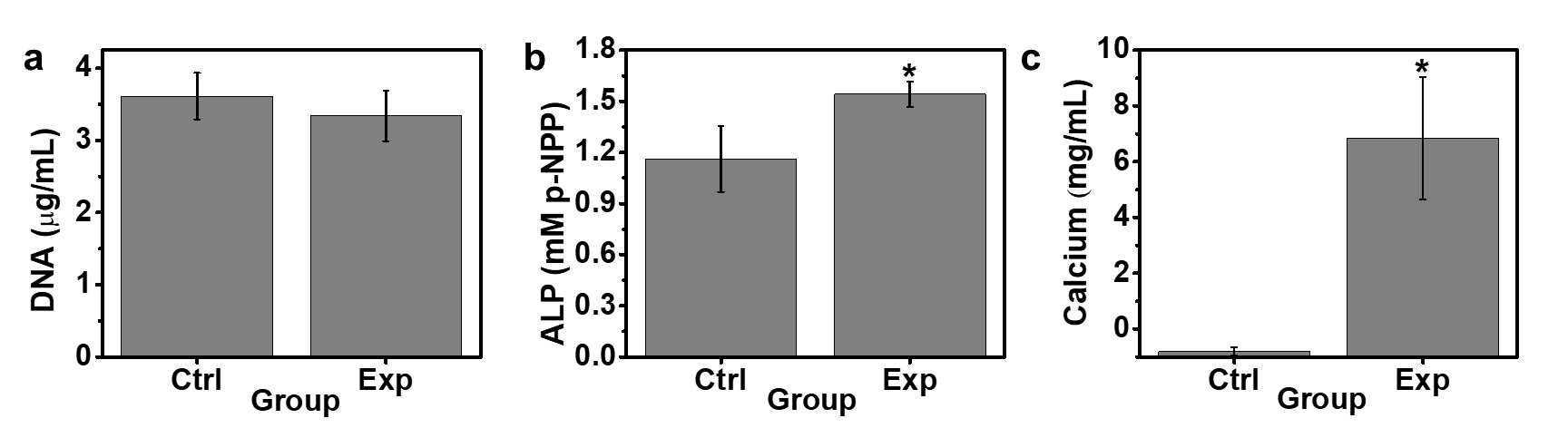


**Figure S16**. Biochemical analysis of (a) DNA, (b) ALP, and (c) calcium contents in Ctrl and Exp constructs at D14. *p < 0.05*.*
